# The dichotomy of human decision-making: An experimental assessment of stone tool efficiency

**DOI:** 10.1371/journal.pone.0327215

**Published:** 2025-07-18

**Authors:** David Nora, João Marreiros, Walter Gneisinger, Antonella Pedergnana, Telmo Pereira

**Affiliations:** 1 The Institute of Archaeology, The Hebrew University of Jerusalem, Mt. Scopus, Jerusalem, Israel; 2 ICArEHB, Interdisciplinary Center for Archaeology and Evolution Human Behaviour, Universidade do Algarve, Campus de Gambelas, Faro, Portugal; 3 Laboratory for Traceology and Controlled Experiments (TraCEr), MONREPOS – Archaeological Research Centre and Museum for Human Behavioural Evolution, Leibniz Zentrum für Archäologie, Germany, Schloss MONREPOS 2, Neuwied, Germany; 4 Institute for Prehistoric and Protohistoric Archaeology, Johannes Gutenberg University, Schönborner Hof, Schillerstraße 11, Mainz, Germany; 5 South Tyrol Archeological Museum, Bozen, Italy; 6 Universidade Autónoma de Lisboa, R. de Santa Marta 47, Lisboa, Portugal; 7 Departamento de Arquelogia, Conservação Restauro E Património, Instituto Politécnico de Tomar, Tomar, Portugal; 8 CGeo - Centro de Geociências, Universidade de Coimbra, Coimbra, Portugal; 9 UNIARQ - Centro de Arqueologia da Universidade de Lisboa, Faculdade de Letras da Universidade de Lisboa, Alameda da Universidade, Lisbon, Portugal; 10 Institute of Evolutionary Medicine (IEM) – University of Zurich (UZH), Switzerland; 11 Institute for Mummy Studies, Eurac Research, Bozen, Italy; Sapienza University of Rome: Universita degli Studi di Roma La Sapienza, ITALY

## Abstract

The physical properties of distinct raw materials, such as hardness, homogeneity, and grain size, have been recurrently suggested as some of the key reasons for human decision-making, namely the selection, production, and use of stone implements in the past. However, little is known, concerning the relationship between stone tools and human behaviour and how this is reflected in the variability seen in the archaeological record. Therefore, investigating stone tools’ properties and performance brings fundamental insights into identifying and understanding the origins of some of the major human technological behavioural traits. In this study, we aim to address this topic by measuring the variability of the properties of lithic raw materials from the perspective of tool use. A controlled experiment was designed to test the mechanical performance with a focus on the efficiency (ratio between effectiveness and durability) of four distinct raw materials (quartzite, dacite, flint, and obsidian). Our study addresses the null hypothesis: *“Edge efficiency does not vary according to the different lithic raw materials.”* Efficiency is assessedby the combination of penetration depth (proxy to measure effectiveness) and edge wear (proxy to measure durability). These two variables were measured, and the results correlated with the physical properties of various raw materials, including hardness and grain size. Our results show significant differences in the efficiency between the different types of raw materials. The outcome demonstrates that the variables by which we test the edge efficiency of lithic raw materials are highly relevant for raw material selection and, consequently, may have been of utmost importance in influencing the decision-aking process of past hunter-gatherers. A decrease in tool efficiency during use may have constrained daily activities, necessitating technological adaptations. This strongly suggests that each raw material used in archaeological contexts to produce blanks *should be evaluated for its efficiency.* In addition, it may be pertinent to extend this approach to other blunt artefactssuch as scrapers, burins, anvils, and hammerstones when investigating aspects of interconnected behaviours such as artefact variability, resource economy, group mobility, and site function. Such choices and decisions are coded in the archaeological record and represent cultural factors that were transmitted through learning and likely triggered the human decision-making process of past hunter-gatherers.

## 1. Introduction

The variability of the Late Pleistocene archaeological record encodes the origin and evolution of key human behavioural traits. Among such variability, stone tools are the most well-preserved source of information to untangle and reconstruct such behaviours. Past stone-based technologies are characterised by the selection and use of different types of rocks, known as lithics or stone artefacts. The production, design, and, ultimately, the use of stone artefacts is often considered to be constrained by the nature (availability or abundance) of each raw material.

In archaeological research, the study of lithic raw material variability often focuses on the traditional dichotomy of so-called high and low-quality lithic raw materials. While researchers focus on the organisation and variability of lithic technologies, a great body of research has highlighted the significance that the “quality” of lithic raw materials imposes on the production, design, and use of stone tool technologies [1]. Quality can be interrelated with several factors since raw material types differ due to “internal” (i.e., fracture predictability, elasticity, toughness, brittleness, hardness, homogeneity, granularity, and isotropy [1–5], and “external” properties (i.e., size, shape, surface regularity, cortex presence, cleavage planes [3,6]. Therefore, when discussing “quality,” one must define how and which proxies are used to quantify such phenomena [7]. Through experimental knapping replications (actualistic experiments), several researchers have shown that not only low availability but also a perceived degree of low-quality raw materials can lead to more expedient (informal) technologies [1,8]. Conversely, stone tool production made from higher-quality raw materials, owing to their high fracture predictability [9–11], tends to be easier to manipulate, leading to more formal or pre-determined technologies [1,8]. On the other hand, the use of different raw materials can have the same effect, where one type of rock can be more or less efficient than another, which raises the quantitative question addressed in this study.

Hence, these terms (high and low quality) have been used to infer suitability for knapping and to assess the decision-making processes marking the evolutionary trajectory of stone tool technologies in the past. This dichotomy is commonly associated with the categorisation of lithic raw materials by grain size. For example, flint/chert (microcrystalline sedimentary rocks) and dacite (igneous rock with heterometric grains) are often defined as fine-grained; obsidian (an igneous glass rock) is typically defined as no-grain. Fine-grained and homogeneous samples have the best properties to be knapped, used, and perform specific tasks [12–14]. By contrast, coarse-grained samples such as quartzite (metamorphic rock very rich in quartz and sandstone grains) are frequently regarded as low-quality raw materials [15–18].

The interplay between rocks of different grain sizes can also be investigated by targeting specific physical and mechanical rock characteristics such as rebound hardness and efficiency [12,19–25]. In 1966, Charles Keller presented one of the first control experiments aimed at testing the efficiency of stone tool edges, noting four main factors affecting an edge: artefact material, a cross-section of the edge, mode of use, and the material upon which the artefact was used. Only the first two are generally recoverable in the archaeological record. In his experiment, the artefact material and the contact material were held constant to isolate the influence of the mode of use (chopping, cutting, scraping). This approach produced “dullness charts” that helped estimate stone tool efficiency: the longer it took for the tool to exhibit physical dullness, the more “efficient” the mode of use. Moreover, Keller observed that using chert rather than obsidian reduced apparent damage, suggesting chert’s greater resilience [21].

Building on this research framework, our work explores this topic by focusing on variability in one of Keller’s four factors: the artefact material (lithic raw materials). Controlled experiments with mechanical apparatuses have been used systematically to measure the physical properties and the mechanical performance of different raw materials, shedding light on both the production and usage of stone tools [12,20,23,25–32]. To some extent, rebound hardness may better capture the rebound of the material rather than simply measuring its static hardness [33]. This approach ties into fracture mechanics, recently applied to distinguish coarse- from fine-grained rock types [20,27,29,32,34]. However, preliminary tests show significant variability even within a single raw material [35,36], often linked to the frequency of microscopic cracks [11] and correlating with grain size [37]. Fine-grained lithologies are generally more effective at absorbing energy than rocks with larger grains [38]. Additionally, rocks with inhomogeneities and bigger cracks typically exhibit lower overall strength [39].

A rock’s ability to withstand strain is commonly assessed through Young’s modulus: a higher value indicates greater resistance to deformation [39]. These metrics aid fracture mechanics studies by helping determine fracture path stability [38]. In this study, we apply the Leeb Hardness Test (LHT), which uses a dynamic impact principle: the rebound velocity (Vrebound) of an impact body is recorded relative to its downward or impact velocity (Vimpact). Harder surfaces yield higher rebound velocities, as they lose less energy to plastic [40].

Our aim is to understand what drove past hunter-gatherers to choose one type of rock over another for critical tasks. Did the classification of rocks by grain size influence both raw material selection and tool use? By applying a set of proxies to quantify the use efficiency of different lithic raw materials, this work attempts to untangle these questions and shed light on an important aspect of past technological decision-making.

Evaluating raw material rebound hardness and efficiency provides insights into the variability of raw materials, which significantly affects stone tool performance. Human decision-making likely considered these variables when selecting lithic raw materials, establishing a hypothetical efficiency value for each. Efficiency is central to human decision-making in utilitarian contexts that involve technological production, design, and use of implements.

Decision-making is a cognitive process that requires the complex integration of multiple information sources. In human decision-making, behavioural and cognitive flexibility or plasticity [41] facilitates adaptation to fluctuating social and ecological pressures. Within Human Behavioural Ecology (HBE), focusing on the principle of optimality in technological decision-making, a hunter-gatherer forager will exploit a resource if its profitability exceeds the anticipated net rate of return per unit of foraging time [42,43]. Such a stance favours the least costly decision because humans have limited energy budgets. Thus, decisions about technological investments (e.g., procurement, production, use of tools) likely depend on a solid knowledge of lithic raw materials, which play a pivotal role in facilitating access to food and other resources [44]. In HBE, raw material availability is often measured in terms of kcal/hour expended to procure and transport the resource, including travel distance and the size/weight of packages. On another level, raw material quality pertains to the resource’s composition and how easily it can be converted or manipulated for technological uses. As Kuhn and Miller [45] state, “this utility comes from the fact that artefacts provide a mechanical advantage that makes certain tasks possible or makes them more time or energy efficient. In other words, although artefacts do not directly supply energy or nutrients to users, by making work more efficient, a usable implement or weapon has the potential to produce a net energy gain for a tool user.”

## 2. Raw material efficiency

In archaeological research, efficiency is measured by the ratio of output to input (Output/Input = Efficiency) [46]. The higher the output (yield) per unit of input (cost), the more efficient the behaviour or technology. Different denominators can be used as a unit to assess efficiency, such as time [47], energy [22], and even raw materials properties [19]. In this paper, these proxies are hardness, effectiveness, and durability [48].

In material sciences, raw material durability is defined as being dependent on its material composition, which can affect strength and appearance. Even though a particular stone may be thought of as uniform, it is still a natural material with varying composition and physical properties. These properties can vary significantly, even for stone removed from slightly different areas of a single quarry or formation. Small variations in some stone artefact types can result in significant differences in durability performance when executing an activity [49–53]. In archaeological studies of stone tools, durability is defined as the physical factor of resistance to abrasion and loss of material during use, which can be measured through experiments [19,20].

In this work, we evaluate the lithic raw material dichotomy from the perspective of tool use with the help of a new method to infer human decision-making through the mechanical behaviour of four common raw materials in the Palaeolithic record. We aim to achieve this by testing via experimental replications the null hypothesis: “*Edge efficiency does not vary according to the different lithic raw materials*.”

## 3. Materials and methods

### 3.1. Raw materials

**Fig 1:**
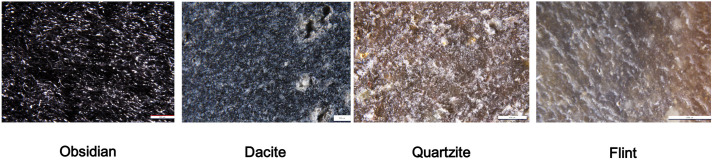
Overview of the lithic raw materials. Microscopic and macroscopic images of obsidian, dacite, quartzite, and flint.

For this experiment, different rock types were selected according to grain size. This study included four raw materials: obsidian (no grain and homogeneous texture), flint and dacite (fine-grained raw materials), and quartzite (coarse-grained raw materials). These were selected from the TraCEr reference collection at MONREPOS-LEIZA Fig 1). Raw materials characterisation is presented and discussed in an MA dissertation work by Asefa [54] and summarized below as follows. Each raw material has a unique geological outcrop reference. In order to ensure data replicability in the experiments performed in the TraCEr laboratory, the same blocks and outcrops of raw materials used by Asefa were selected for this study. Obsidian and dacite [55,56] were collected in Armenia, quartzite in Germany, and flint in Belgium [57,58]. Flint samples consist of heterogeneous nodules with a light-to-dark colour and lighter-coloured inclusions (<0.5-1 mm) with different silicification degrees in some areas of the nodule [57]. The petrographic analysis identified microcrystalline quartz and calcite within the flint. XRD results showed that quartz made up approximately 98.1% of the composition, with evidence of calcite. SEM-EDS elemental mapping revealed the presence of Si, Ca, P, and C, with silicon being predominant, aligning with the quartz-rich nature. The association of calcium with carbon suggested the presence of calcium carbonate, likely from shell structures, while the presence of calcium and phosphorus indicated possible apatite, potentially from organic materials.

Quartzite was gathered from the Rhine River (Germany) basin. It displayed an inequi-granoblastic texture, predominantly composed of quartz with minor muscovite. XRD analysis confirmed that quartz constituted about 97.2% of the mineral composition, with muscovite present as a secondary mineral. SEM-EDS mapping detected elements such as Si, K, Al, P, Fe, Ti, Mg, Ca, and Na, with silicon dominating, consistent with the high quartz content. The presence of aluminium and potassium indicated the occurrence of muscovite.

Obsidian samples are originally from Armenian outcrops and consist of a light black and translucentcolour. Due to its glassy texture, petrographic examination did not reveal distinct mineral phases except for occasional amphibole and plagioclase crystals. SEM-EDS analysis confirmed the absence of crystalline phases, with the material predominantly composed of Si and Al, and minor peaks of Na, K, Fe, and Ca. This composition is consistent with the felsic nature of obsidian.

Dacite, also from Armenia, presents a dark colour with some microcrystals of quartz inclusions. Petrographic examination revealed a porphyritic texture characterised by phenocrysts of plagioclase feldspar and quartz, with minor ferromagnesian minerals such as biotite. The groundmass exhibited signs of weathering, including the presence of calcite. XRD analysis quantified the mineral composition as approximately 54.7% albite and 42% quartz, with trace amounts of biotite and calcite. SEM-EDS elemental mapping identified silicon (Si), aluminium (Al), potassium (K), sodium (Na), calcium (Ca), iron (Fe), titanium (Ti), and phosphorus (P), corroborating the dominance of quartz and albitic plagioclase, along with minor K-feldspar, ferromagnesian phases, and apatite.

These comprehensive analyses elucidate the mineralogical and chemical characteristics of each raw material, providing valuable information for understanding their properties and potential applications.

### 3.2. Leed rebound hardness

Hardness is an important physical property of rocks, directly related to their fracture mechanics [19,20]. In our study, focusing on the perspective of tool use, the Leeb rebound hardness of each fine and coarse-grained raw material was measured and evaluated as the main physical property. To measure the hardness of the four lithic raw materials, the Leeb rebound hardness Test (LHT hereafter), Equotip Leeb Impact Device C with probe serial number IC51-004-0185 was used (see S1 for all equipment specifications and acquisition settings). This instrument is used in industry, but the configuration software allows calibration for different hard materials, so a configuration for lithic raw materials was developed. The configuration is determined by the outline dimensions of the sample (length, width, thickness, and roughness scale) and the weight. To ensure the accuracy and internal variability of the tests, all samples were previously inspected and registered for macroscopic impurities to determine if any deviation could affect the result. The Leeb Impact Device was used in an accurate vertical position [59], and since the LHT required specific minimum properties, samples were placed on a base (granite slab) and connected with a layer of reversible coupling paste. To reduce intra-sample variability and test internal variability, each rock was measured ten times at different locations (randomly sampled on the samples’ surface). Measures closer to the edges and defects were avoided during testing. The results were exported to a.csv file for further data analysis, and a.pdf report for each sample was exported with the Leeb instrument reference and additional information (see S2). On this topic, Ghorbani et al. [60] suggested a qualitative classification into six groups of the Leeb scale from extremely soft to extremely hard, which we will use for conformity qualitative classification of the samples. Descriptive statistics were calculated for the hardness values for comparison within and between samples. All data analysis and plotting were processed with the R open-source software (see S2 for detailed info on used packages and software versions, scripts, and raw and processed data).A research compendium using the rrtools package by Marwick et al., (2019) [61], including detailed info on used packages and software versions, raw and processed data is available here: https://github.com/jmmarreiros/noraetalstonedura2025, under the MIT license, data under CC-0, and figures under CC-BY (see further details in Marwick, 2017 [62]).

### 3.3. Contact material

**Fig 2:**
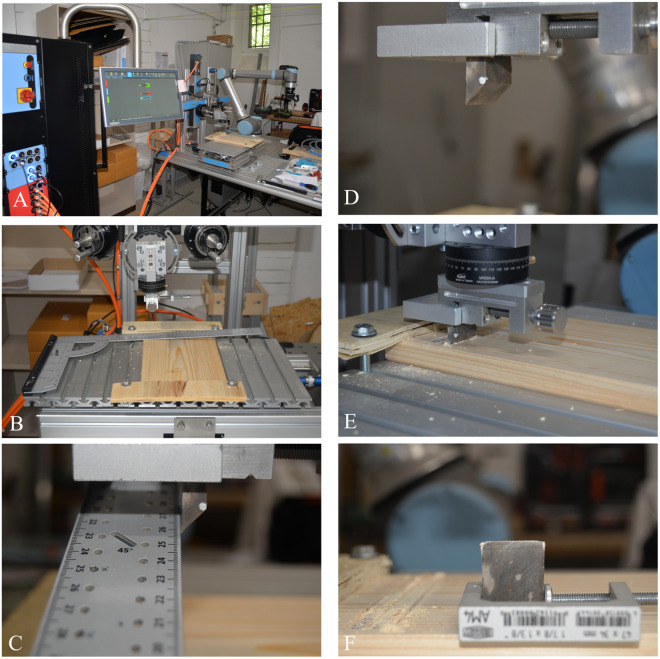
Experimental setup of the SMARTTESTER® device used for cutting experiments. (A) Overview of the setup; (B) Alignment of the plank; (C) Alignment of the sample; (D) Sample ready for performing the experiment; (E) Sample performing the experiment bidirectional linear movement; (F) End of the Experiment.

A single plank of pinewood was selected as a contact material to be used in all experiments, using different areas of contact. The selection of this contact material and the use setup took into consideration the previous protocol by Abrunhosa [15] and Pereira [29]. Previous works noted that when performing their experiments, the tool edge would fall between the growth rings. Therefore, the experiment should replicate a perpendicular cutting movement of the large boards with a full range of angles of the log’s rings, also known as the log’s “grain”. For commercial purposes, wood hardness is universally tested using the Janka hardness test, which involves measuring the force required to push a steel cylinder with a diameter of 11.28 millimetres (resulting in a circle with an area of 100 square millimetres) into the wood to a depth of half the cylinder’s diameter. The output data are exported in pressure units, which are then converted to kgf (kilograms force) [63,64]. Pinewood plank was obtained from the Bauhaus commercial bricolage centre in Germany. European Pinewood has an average density of 550 kg⁄ m3 and a moisture content of 12–15% [55]. In order to perform the desired cutting movement, we used flat-sawn wood plank (Fig 2).

### 3.4. Sample preparation

**Fig 3:**
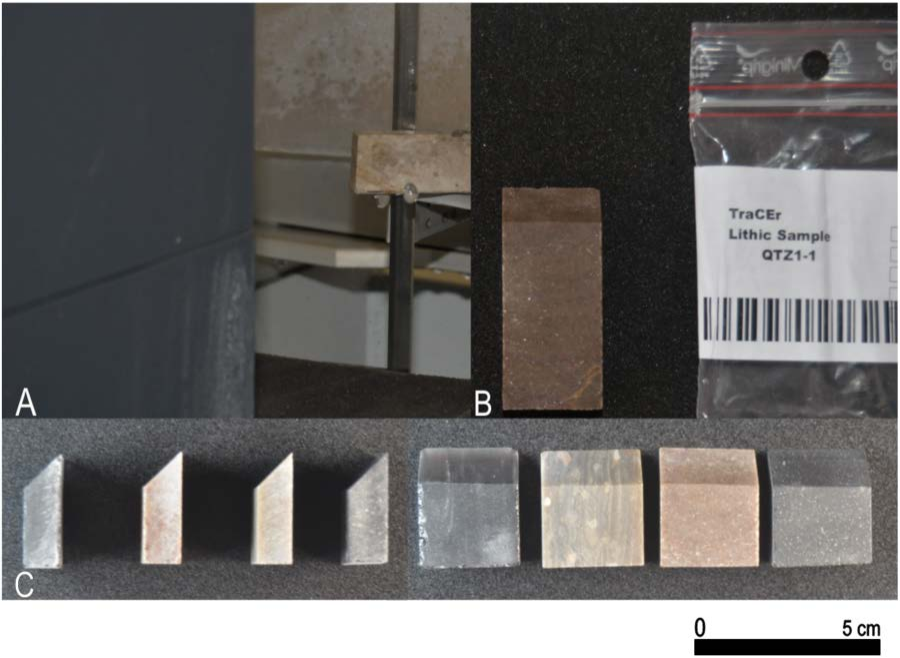
Preparation of lithic samples. (A) Standardised cutting of blocks into dimensions (30 mm x 25 mm x 10 mm) with a 45° edge. (B) Processed samples labelled and packed. (C) Array of prepared lithic samples from all raw materials.

We produced a standardized morphology and shape for all lithic samples to avoid the coexistence of differences in key geometric features that are known to affect tool use, such as edge angle, edge length, and edge morphology [26,65,66]. To ensure raw material invariability within each rock type, a single nodule from each was used to produce all experimental samples (3 samples per nodule were taken from 4 different nodules to produce 12 specimens). To ensure uniformity in the morphology and length of the edges, all specimens were saw-cut with a length of 30 mm, a width of 25 mm, a thickness of 10 mm, and a 45° unifacial edge (representative of the active edge, see [67]) by a diamond band saw (Fig 3). Each piece was assigned a unique ID and a bar code, and weighed, using a balance accurate to the first decimal digit.

Following preparation, all samples underwent a standardised cleaning protocol. This cleaning regime was sufficient to remove all residues from the wood-cutting process. The samples were rinsed in tap water, cleaned with industrial alcohol and acetone, and left to dry at room temperature.

### 3.5. Imaging observation, documentation, and analysis

**Fig 4:**
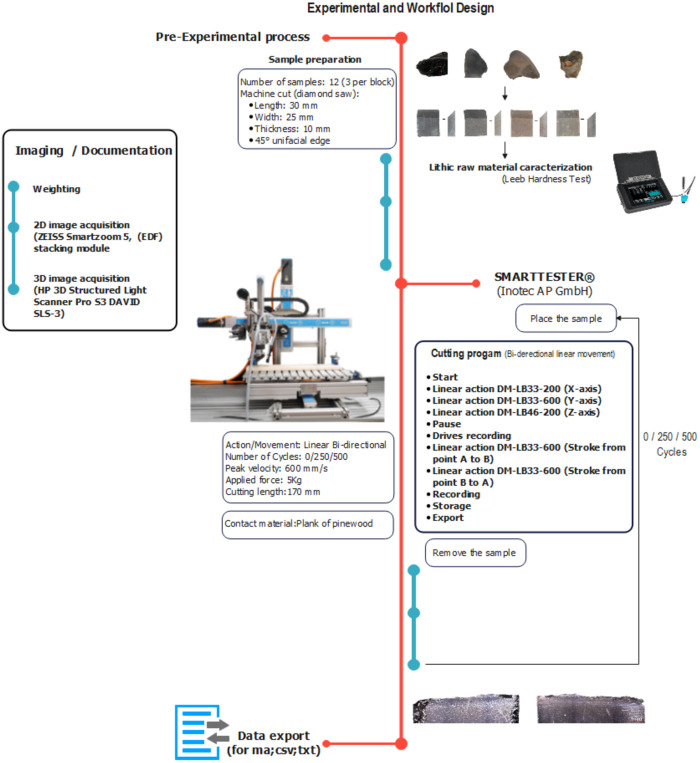
Experimental workflow and design. This includes pre-experimental preparation, imaging, raw material characterisation (Leeb hardness), and cyclic cutting tests, which culminate in data collection.

In this experiment, we performed a sequential experiment in 3 stages, and each sample was recorded at stage 0 (before the experiment), stage 1 (125 cycles), stage 2 (125 cycles) and stage 3 (500 cycles after the experiment was complete). The documentation protocol was performed the same way for each stage of the experimental design (Fig 4): cleaning of the sample, weighing, 3D scanning, and digital microscope imaging. The 3D documentation was done with a portable HP 3D Structured Light Scanner Pro S3 DAVID SLS-3. For image comparison, a digital automated microscope ZEISS Smartzoom 5 (equipped with a PlanApo 1.6/0.1x objective and an integrated segmented LED ring light) was used to image larger areas with low magnification. To acquire the images, a program (ZEISS Zen Core) was used, with the image Extended Depth of Focus (EDF) stacking module to create in-focus images. Three surfaces were documented, the two main surfaces (front/back side) and one lateral side. A digital camera (Nikon DSLR camera, model D610 with a Nikon AF-S Nikon 50 mm f/1.8g lens) was used to capture a broad morphological representation of the samples (see S1 for all equipment specifications and acquisition settings).

### 3.6. Experimental Design

By attempting to differentiate lithic raw materials, we introduce an experimental design to test and quantify the efficiency of the active edge of each stone tool from four lithic raw materials divided into categorical groups – quartzite, flint, obsidian, and dacite. These were used in a 3-stage bidirectional cutting movement: stage 1 (cycle: 0–125, total of 250 strokes), stage 2 (cycle: 126–250, total of 250 strokes), stage 3 (cycle: 251–500, total of 500 strokes); all stages combined in a total of 1000 strokes (Fig 4). Efficiency was calculated by the ratio between the degree of penetration depth (effectiveness) and edge wear (durability). This approach builds on previous methods and protocols [15,25,27,29,31]. This experiment is categorised as a second-generation experiment (sensus Marreiros et al., 2020 [68] and does not directly attempt to reproduce human actions or real-life activities but rather aims to test the resources used by humans in the past. Instead, expert systems (mechanical devices) [69] are harnessed to develop fundamental principles by isolating, regulating, and evaluating the cause-effect relationship of variables, from which a more detailed and accurate understanding of the process can be derived.

**Fig 5:**
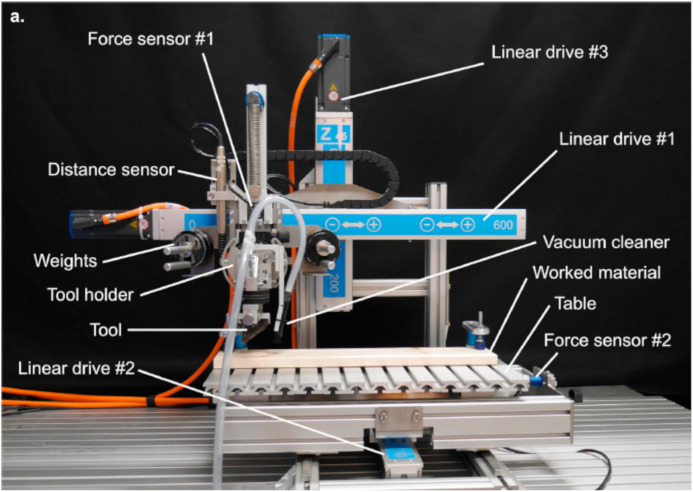
Detailed view of the SMARTTESTER® device.

We used a modular mechanical device (SMARTTESTER®, manufactured by Inotec AP GmbH with modifications made by one of the authors WG) (Fig 5) (see details in [70]. The SMARTTESTER® system is composed of linear drives and several sensors connected to a programmable centralised controlling/computing unit.

This mechanical equipment allows for the control and measurement of a large number of variables according to the template, such as peak velocity, acceleration, applied force (via dead weights), angle of work, number of movements, position, friction, and travel distance.

The Inotec Smarttester’s versatility allows the exploration of different setups (linear, rotary, percussion, and oscillating [70]. For this experiment, the linear setup was used, which consists of three linear drives mounted in this configuration to move the tool and worked material in three directions: linear drive #1 moves the tool along the X (horizontal) axis, linear drive #2 moves the worked material along the Y (horizontal) axis, and linear drive #3 raises and lowers the tool along the Z (vertical) axis (Fig 5) [70].

### 3.7. Measuring efficiency

In this study, lithic raw material efficiency was measured using the following equation: $Edge\;efficiency=\frac{Effectiveness\;}{Durability\;}$*.* In the experimental design, effectiveness (the degree to which something is effective/done [48], is represented by penetration depth (PD), and durability (the degree to which deterioration occurs or volume loss) is represented by edge wear (Ew). Effectiveness (penetration depth) is measured by the depth sensor, which measures penetration depth for each stroke, then compiles it into a cycle.

**Fig 6:**
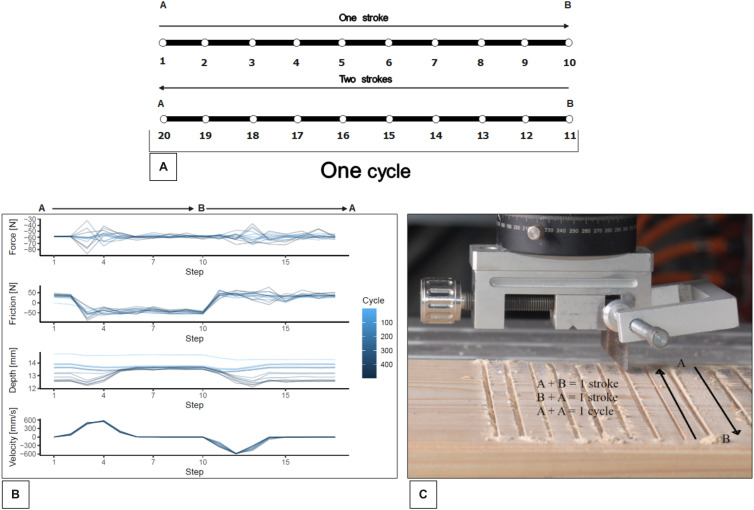
Cutting cycles explained. (A) A single cycle involves forward and reverse strokes. (B) Force, friction, depth, and velocity data recorded during experiments highlight performance differences among raw materials. (C) Cycle representation.

From each stroke, the Smarttester takes twenty measures. These same measures were then recorded and saved to be imported for quantification and visualisation for every stroke and then grouped for the representation of each lithic raw material (Fig 6).

Durability is measured through the Edge wear (Ew) as a proxy. Here, we consider edge wear based on tribomechanics definitions, see Table 1. Edge wear (also mentioned as edge attrition [20] is the process of detachment of material between the active sample and the contact material [71–74]. The detachment of material may be due to factors such as a) adhesion + fracture, b) abrasion + fracture, and c) fatigue + fracture [71,72]. On this basis, use-wear (or traces of wear and use) results from two physical processes: edge friction and edge wear [73–76]. Thus, in this study, the degree of material loss (a proxy for measuring durability) from the edge is classified as edge wear. Hence, durability is expressed as the sum of material loss from the edge measured on each before and after use. The different experimental stages were compared using the software CloudCompare with the computation tool (Cloud-to-Mesh, C2M). The distance between C2M is computed as the absolute Hausdorff distance, hereafter aHd (also called Pompeiu–Hausdorff distance) [25,27,77–79]. To reduce the chance of calculating what could be error accuracy caused by the scan acquisition or alignments error, we have applied a 0.5 mm error threshold (the 3D equipment’s error threshold) to classify the cloud distances.

**Table 1:** Tribological and Archaeological definitions of Friction and Wear.

Concepts	Tribology definition	Archaeological definition
**Friction** **(Edge Friction)**	“*The force known as friction may be defined as the resistance encountered by one body in moving over another. This broad definition embraces two important classes of relative* *motion: sliding and rolling.*” [71–72].	“*Wear as a physical process is divided into two basic types (...)The second type comprises the less noticeable manifestations of deformation in the tool, which we can call micro-deformation. The latter is observable in those very frequent cases when wear arises from friction between the tool and the object of the work.*” [73–74].
**Wear** **(Edge Wear)**	“*Wear is the process of detachment of material from one surface. It is different from friction in the sense that it is not taking place at a certain moment but during a time period when the surfaces are in moving contact*.” [71–72].	“*Wear as a physical process is divided into two basic types. The first type is the very rough form of deformation of a tool during the work. This comprises all kinds of alteration that arise in the course of blows that damage the working part by the dislocation of comparatively large pieces, discolorations, shatter, creation of scars, dents, notches, cracks, and so on.*” [73–74].

The colder colours depict low distance values (i.e., the target specimen is a close match to the reference specimen), and progressively warmer colours depict increased distances between the specimens (i.e., greater variation). The distribution of the absolute distances is also visualised through a histogram. Our comparison included the sample without any wear (in stage 1) and a subsequent comparison at every stop between the selected stages of the experiment. Traces of wear were only documented through a low-magnification digital microscope. After comparison, the data generated from each computation was exported and combined in a single.csv file and imported into an R script for the statistical analyses (see S3). For a more detailed description of the material and methods used in this study please check the protocol at dx.doi.org/10.17504/protocols.io.bp2l6djq1vqe/v1.

## 4. Results

### 4.1. Raw material hardness

**Fig 7:**
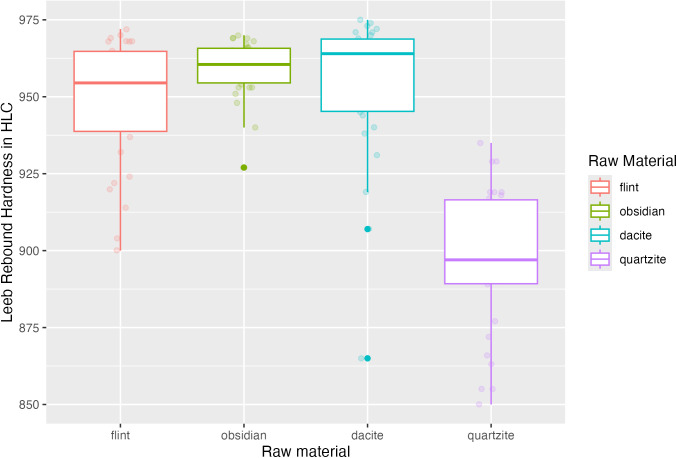
Leeb hardness values of flint, obsidian, dacite, and quartzite. The boxplot illustrates material hardness variability.

In this study, the selected lithic raw materials show different mineralogic and petrographic categorisations. Fine-grained, including flint and dacite, coarse-grained, quartzite, and obsidian, with no grain and homogenous texture. As a physical property of the different types of rocks, the Leeb rebound hardness was assessed on all samples from the four raw materials in this experiment. The boxplot shows the values distribution and comparison between all collected data samples (Fig 7).

While evaluating hardness as an element of the traditional signature of coarse and fine-grained lithic raw material categories, it is possible to see a clear division within the proposed classification. In fine-grained material, flint registered higher variability (mean range between 936–965 HLC). These values fall into the category of hard and extremely hard rock types [60] and are similar to the ones available for obsidian (mean range between 951–965 HLC). However, the latter rock type presents a cluster of values representative of extremely hard classification, according to Ghorbani [60]. Dacite registers a range of (932–965 HLC) described as extremely hard. On the other hand, coarse-grained material has a wider variability between the measurements. While quartzite means values vary between 883 and 907 HLC, it is classified as moderately hard. Based on this range of values, we can see a division between quartzite and the other three raw materials. Data dispersion also indicates that fine-grained materials present more concentrated data than coarse-grained materials, showing more dispersed values.

### 4.2. Penetration depth (effectiveness)

**Fig 8:**
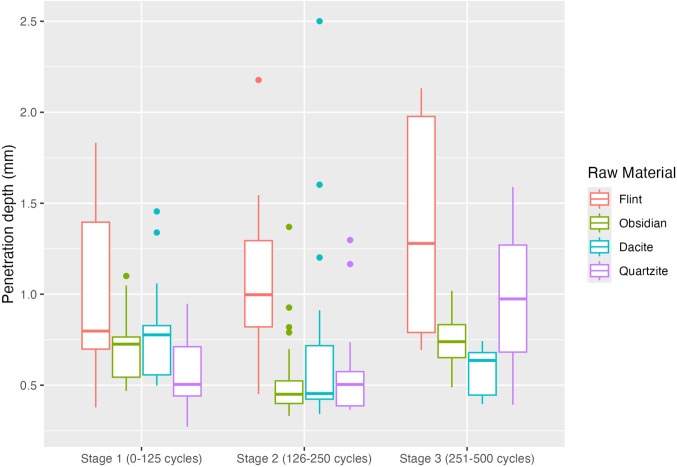
Penetration depth (mm) across experimental stages for all raw materials. High effectiveness (High values of penetration depth), Low effectiveness (Low values of penetration depth).

Penetration Depth (PD) is used to assess the effectiveness of all samples during the experiment. All samples reached the last stage of the experiment, which consisted of 3 experimental stages. Figure 8 shows the results of the PD for each stage organised by lithic raw material. For a simple and easier visualisation, the boxplot represents all the samples grouped as part of the respective lithic raw material (see details on each sample record in Table 2 and S2.

**Table 2:** Measurements per sample and cycle.

Raw material	Cycle	Sample id	Force	Friction	Velocity	Depth	Edge wear (aHd)	Hardness (HLC)
Dacite	0-125	DAC3−2	−58,669	−13,166	1,993	0,921	38	942
Dacite	125-250	DAC3−2	−58,427	−14,322	0,285	1,202	23	942
Dacite	250-500	DAC3−2	−58,564	−0,894	−0,126	0,729	14	942
Dacite	0-125	DAC3–4	−58,493	9,873	2,658	1,455	8	965,4
Dacite	125-250	DAC3–4	−58,043	−4,877	1,406	2,501	72	965,4
Dacite	250-500	DAC3–4	−58,234	11,772	2,627	0,626	13	965,4
Dacite	0-125	DAC3–6	−58,781	8,233	2,992	1,339	48	963,9
Dacite	125-250	DAC3–6	−58,968	1,313	1,813	0,768	1	963,9
Dacite	250-500	DAC3–6	−58,959	4,855	2,146	0,742	21	963,9
Flint	0-125	FLT10−2	−59,010	22,002	2,677	1,833	41	965,6
Flint	125-250	FLT10−2	−58,893	25,835	2,039	2,177	53	965,6
Flint	250-500	FLT10−2	−58,989	−4,392	0,562	1,054	80	965,6
Flint	0-125	FLT10−5	−58,649	−7,211	0,668	0,720	5	936
Flint	125-250	FLT10−5	−58,600	20,159	0,934	1,506	56	936
Flint	250-500	FLT10−5	−59,422	20,277	1,824	2,132	2	936
Flint	0-125	FLT10−6	−58,716	4,715	2,637	0,996	33	945,4
Flint	125-250	FLT10−6	−58,603	12,981	2,185	1,545	19	945,4
Flint	250-500	FLT10−6	−58,783	1,119	2,630	1,386	21	945,4
Obsidian	0-125	OBS4−4	−58,301	2,226	2,625	1,048	44	965,7
Obsidian	125-250	OBS4−4	−58,957	2,297	1,880	0,819	46	965,7
Obsidian	250-500	OBS4−4	−58,515	3,587	0,902	0,973	52	965,7
Obsidian	0-125	OBS4–5	−58,666	4,180	2,645	1,006	47	959,8
Obsidian	125-250	OBS4–5	−57,728	−4,129	0,592	1,370	76	959,8
Obsidian	250-500	OBS4–5	−57,934	−2,437	0,676	1,018	69	959,8
Obsidian	0-125	OBS4–6	−58,667	6,592	3,028	1,100	56	951,8
Obsidian	125-250	OBS4–6	−58,810	−6,261	0,881	0,926	15	951,8
Obsidian	250-500	OBS4–6	−58,378	2,797	1,022	0,756	106	951,8
Quartzite	0-125	QTZ1–2	−58,811	−4,594	2,588	0,947	47	907,7
Quartzite	125-250	QTZ1–2	−58,857	4,512	2,634	1,298	4	907,7
Quartzite	250-500	QTZ1–2	−58,524	8,120	2,274	1,589	42	907,7
Quartzite	0-125	QTZ1–5	−58,915	−3,301	2,041	0,475	111	899,3
Quartzite	125-250	QTZ1–5	−58,959	−2,350	3,072	0,736	2	899,3
Quartzite	250-500	QTZ1–5	−58,864	0,932	2,602	1,561	12	899,3
Quartzite	0-125	QZT1−1	−58,569	1,058	2,473	0,632	25	883,3
Quartzite	125-250	QZT1−1	−59,037	−2,626	1,171	0,733	5	883,3
Quartzite	250-500	QZT1−1	−58,771	5,991	2,802	0,734	30	883,3

From stage 1, flint was the raw material that reached deeper, almost 2 mm. Obsidian and dacite showed a similar performance, reaching 1 mm of PD. In this cycle, quartzite has a low penetration, not even reaching 1 mm. In the second stage (126–250 cycles), dacite reaches around 2.5 mm, followed by flint with approximately 2.3 mm. Although dacite had reached a deeper value, flint is more consistent than dacite. For obsidian and quartzite, only a few strokes reached a depth of 1.5 mm, with most strokes resulting in an average depth of about 0.5 mm. In the first two stages, each involved a total of 250 strokes, while the third stage (251–500 strokes) saw an increase to 499 strokes. This led to a significant depth for the flint samples, reaching approximately 2.3 mm, with an overall arithmetic mean of more than 1 mm across all experiments. For quartzite, the penetration depth (PD) increased to nearly 1 mm, with a maximum of over 1.5 mm. Obsidian, however, maintained consistent PD values throughout, and dacite remained below 1 mm in this stage.

**Fig 9:**
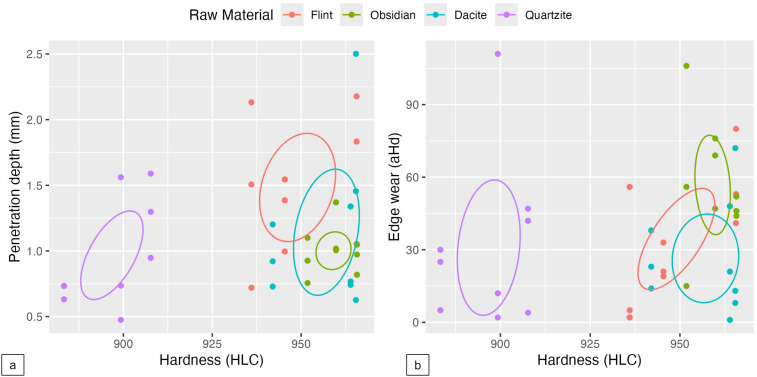
Scatterplots comparing raw material (a) hardness with penetration depth and (b) edge wear.

To inspect the relation between the dichotomy of the grain sizes concerning the values of PD and hardness, data is presented in a scatter plot (Fig 9-a). From the data distribution and visualisation, we can see a clear division between raw materials, with one set of overlapping values (dacite/flint/obsidian). The data presented shows us that the fine-grained raw material (dacite) is the one to reach higher penetration depth values, and also the ones with higher values of HLC (Fig 9-a), which, according to Ghorbani [60], is considered an extremely hard material. Samples referenced as fine-grained have higher HLC values and presented more consistent higher values for penetration depth. However, flint presents a more consistent mechanical behaviour for penetration depth, even with more variability in the HLC values. Obsidian, on the other hand, registers higher HLC values but, in comparison with flint, does not have higher PD values. However, there is one sample from this group that, during the experiment, had minimal penetration.

### 4.3. Edge Wear (durability)

**Fig 10:**
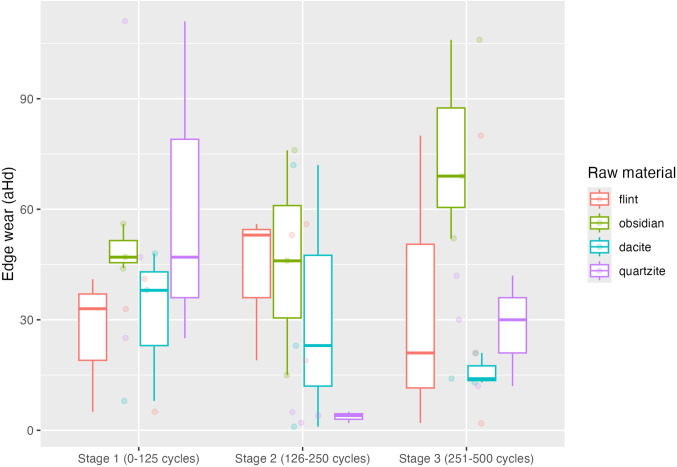
Edge wear across experimental stages for all raw materials. High Durability (Low edge wear values), Low Durability (High edge wear values).

Edge wear is represented in Figure 10 in the same way as PD, all samples were clustered as representative of each raw material in each cycle. The first stage shows us a successive series of detachment coming from the quartzite samples, with values overpassing the 90 Hausdorff distance between two points. Obsidian followed by dacite registers a lower distance, below the distance values of 60. C2M analysis for flint generated a range of distance values between 0–40. As for the second stage (cycles:126–250), quartzite stands out as an almost stagnation stage, with distance values between the two meshes ranging from 0 to 5 Hausdorff distances. Obsidian with an increased distance range from 0 to 76. Dacite registers values from 0 to 71. Flint had an increased distance, reaching 57 values of distance. At the last stage (cycles: 251–500), dacite did not overpass a distance value of 30, followed by quartzite, which had an increase closer to 45. Flint, as it constantly progresses in distance at the last stage, has a range of distance values between 0 and 80. As for obsidian, the range reaches a distance value above 105.

Fig 9-b explores a visual analysis of edge wear, hardness, and grain size. Our independent variable is the division based on the HLC values. In this scenario, the softer material, represented mostly by quartzite as a coarse grain rock, shows lower distance values, meaning a lower frequency of detachments. Interestingly, dacite, characterised as a fine-grained with higher values for the HLC, delivered the same outcome on the edge wear as quartzite with distance values between 0 and 20. As for fine-grained material, there is a higher variability, but the visual data indicates that the higher the values of HLC, the higher the edge wear values, even though there are also lower distance values.

### 4.4. Raw material efficiency (Effectiveness/Durability)

This section aims to explore each stage’s results regarding the relationship between effectiveness and durability, which shows the efficiency value of the two groups.

#### Stage 1 (cycle:0–125).

**Fig 11:**
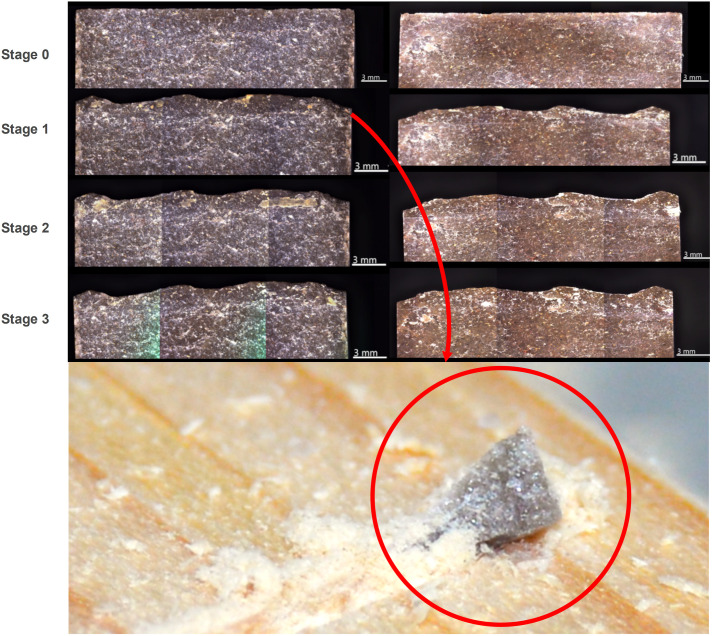
Micro-detachment on the edge of a quartzite tool after use.

Quartzite, as a metamorphic rock (coarse-grained), had a series of fragmentations in an earlier use stage. This result could be explained by LHT results, showing that quartzite is significantly less hard when compared to the rest of the lithic raw materials. Also, when comparing the minimum and maximum hardness values, we see the lowest measures for hardness. In this first stage, all three quartzite samples show greater wear in the Hausdorff distance values. Although edge wear happens at an early stage of use in all raw materials, it seems to occur more frequently on quartzite. Based on these observations, our interpretation is that the earlier fracture is possibly due to the larger quartz grain size that characterises the matrix of the quartzite. Larger particles are easily detached during the first 250 strokes. This wear pattern is also visible in the debris (microscopic detachments and fragments) of the quartzite carved into the pinewood (Fig 11). From a tribological perspective, a possible relation can be made between the fracture outcome after 250 strokes and the two deformation types that occur on the quartzite samples: a) deformation through pressure among the grain margins, which is perpendicular to compressive forces, b) plastic inter-crystalline deformation, where the applied forces provoke dislocations in the grain structure [80].

Moreover, our results show that the obsidian samples remained unaltered/unchanged during the first strokes. Similar to flint and dacite, all samples show a small proportion of edge wear. Regarding PD, the results from the absolute values of all raw materials in stage 1 show that quartzite was the raw material that did not penetrate as deeply as the other three, probably, and as discussed above, due to the high fragmentation of the edges. Inverse observations were made on the dacite and flint samples. The latter registered higher values for PD into the pinewood, while the edges did not show extensive damage.

Based on these results and observations, we argue that in this first stage (250 strokes) fine-grained raw materials are the most efficient ones, showing a lower degree of fracture by edge wear associated with higher penetration values.

#### Stage 2 (cycle: 126–250).

A clear difference can be observed in the next stage (126–250 strokes), considering that the same number of strokes was applied to the same samples as in stage 1. Here, quartzite is replaced by obsidian and dacite, with the highest values for edge wear. Quartzite samples, as a consequence of the high edge wear in the first stage, resulted in irregular and dull edges (S6), which seem to be more “stable” after the initial stage of fragmentation. Unlike quartzite, flint shows a continuous increase in edge wear.

The same relationship is observed for the PD values in the first stage. Flint and dacite are the lithic raw materials that show do reach higher penetration values, followed by significant damage to the edge. In this stage, obsidian shows a similar pattern to the quartzite samples in the previous stage since it shows low PD values followed by high edge wear values. In this experimental stage, all samples hold a modified edge through use. However, our experiment aims to test the performance between different lithic raw materials and not the different types of edge modification, so we kept the modified edge as the result of the use on previous stages in a continuum until the last experiment stage.

At the end of this stage, we can observe that the coarse-grained raw materials are, in fact, more efficient than fine-grained ones since they do not fracture as much and still record measurements of higher penetration depth.

#### Stage 3 (cycle: 251–500).

From stroke 251–500, the most profound difference is seen in the dacite samples, where there is a decrease in PD values, followed by low edge wear values. Regarding quartzite, once the edges of the samples are stabilised, the fragmentation process resumes. However, this is now accompanied by an enhancement in edge efficiency as higher PD values are achieved. As for dacite, it appears that in this stage the plateau of performance (low PD and low Ew) is reached. For the other raw materials, both flint and obsidian displayed high edge wear in the last cycle of the experiment but showed differences in terms of PD values. Obsidian displays lower values of penetration, which are linked to the high fragmentation and brittleness of this raw material. This pattern was also observed in the first cycle on quartzite which showed high edge wear values, followed by low PD in the contact material.

Still, in stage 3 (stroke 251–500), flint registered a different pattern from the previous stages. For one of the samples, due to a micro-detachment on the edge (see S4), the penetration depth increased. This phenomenon is very interesting because it shows a clear difference between obsidian and quartzite. Since the occurrence of micro-detachments does not result in higher PD values, further data are needed to support this relationship statistically. In all the other stages, flint had the most homogeneous performance, as it did not break as much as would be expected because it is a fine grain.

**Fig 12:**
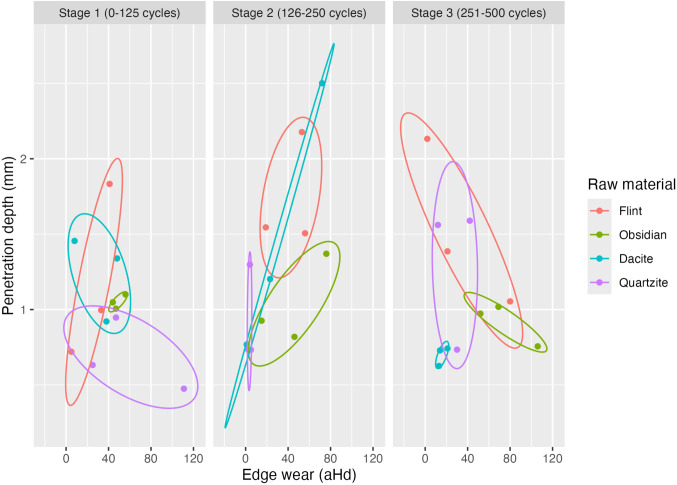
Penetration depth vs. edge wear at different stages. Scatterplots demonstrate the trade-offs between lithic raw material efficiency.

From the scatter plot of the last cycle (Fig 12), we can observe that quartzite, along with flint, becomes more efficient since it shows higher PD values and lower Ew values.

In general, if we refer individually to each parameter in terms of effectiveness, flint is the most penetrating raw material measured in accumulated millimetres, followed by dacite, quartzite, and obsidian, which are the least effective ones. As for durability, we find the same cluster, flint and dacite are the most durable materials, whereas quartzite and obsidian have higher wear-by-distance values, so they are less durable.

**Fig 13:**
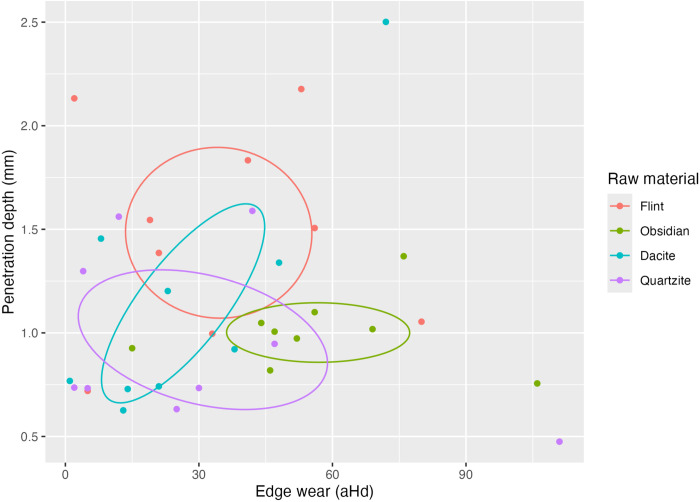
Combined efficiency analysis. All Materials at all stages are evaluated for cutting effectiveness and edge wear (these are the absolute values for of all cycles).

Figure 12 shows a weak correlation between penetration depth and edge wear. However, when examining high-efficiency samples in the top left corner of the scatterplot (4th quartile), characterised by low penetration depth and low edge wear, we find that flint and quartzite consistently exhibit high efficiency. This observation rejects the null hypothesis that edge efficiency does not vary significantly according to different lithic raw materials. Instead, it supports the alternative hypothesis that efficiency varies with the type of raw material used under certain conditions. (Fig 13).

## 5. Discussion

Stone artefacts vary in their raw material, technology, typology, and functionality. However, these attributes always begin with the selection of available resources in the landscape, guided by their suitability for intended tasks. Since Keller’s [21] archaeologists have attempted to disentangle the concept of variability from controlled experiments, seeking parsimonious explanations for key archaeological observations: a) Why did prehistoric people select, manipulate, and use diverse lithic raw materials? b) Can we use stone artefacts variability to infer decision-making processes from past hunter-gatherer behaviour? c) What dictates decision-making when multiple lithic raw materials are available?

Nelson [81] suggested that technological strategies are not fixed, rather they are situational behaviours dependent on environmental, social, and cultural factors. Factors like resource acquisition, mobility, and cost efficiency shape technological strategies at particular times and places. Various strategies are associated with time management and cost control, thus enhancing technological efficiency [82]. Accessible raw materials facilitate expedited production, requiring minimal configuration and infrequent repairs. Conversely, investing time and energy into raw materials enables the production of durable tools that can be regularly maintained [31,83,84]. Such behaviours manifest archaeologically through variations in raw material organization and the balance between local and imported materials [85,86]. As highlighted in our study, from the perspective of the HBE and present on ethnographic data [42], past hunter-gatherer decisions regarding technological investments (procurement, production, and use of stone tools) relied on knowledge about lithic raw material efficiency, allowing optimal resource acquisition. However, ethnographic observations and recent archaeological studies indicate that humans do not always pursue optimal technologies. Instead, technological strategies may be influenced by non-functional selective pressures (such as mobility) or cultural-historical constraints [82]. Although cultural-historical factors cannot be directly discerned from the archaeological record, testing the function and use of artefacts can help identify the reasons behind technological shifts.

Therefore, archaeologists need to develop proxies to quantify each source’s net rate of return, in this case, lithic raw material variability and tool performance. When measuring the efficiency of lithic raw materials, two main proxies should be considered: effectiveness and durability [12,21,22,24,25,27,31,83].

Our study experimentally evaluated the efficiency of different raw material categories by measuring edge efficiency. This was assessed by replicating cutting-like movements on pinewood under controlled conditions, specifically measuring penetration depth (PD) and edge wear (Ew). Across three experimental stages, efficiency varied notably among the four rock types. Fine-grained, coarse-grained, and no-grained materials showed distinct efficiency levels at different stages (Fig 12). Flint exhibited constant edge efficiency, characterised by increased effectiveness but decreased durability, suggesting higher mean proportional penetration depth with prolonged use. Dacite performance plateaued after the initial stages, stabilising with minor PD and Ew variations. In the last stage of the experiment, dacite shared the same pattern as quartzite between the first and the second stage, resulting in high values of PD and Ew, followed by a stabilisation of the edge (e.g., low degree of damage or material loss). Quartzite showed high variability in durability but gradually increased effectiveness. Obsidian behaved similarly, with effectiveness linked to edge deterioration (micro-detachments acting as self-resharpening). Increasing edge wear correlated positively with penetration depth, likely influenced by physical features like sharpness or brittleness [22,24,87,88]. In our experiment, we focus on one edge morphology to avoid adding more variables and to have minimal control over edge sharpness since Collins [26] had already shown that different edge morphologies are likely to have different efficiency outcomes. Sharpness relates directly to the tip radius of the edge [22,87]. Although we think different raw material edges at the microscopic level are morphologically distinct, e.g., the obsidian molecular-level sharpness contrasts with quartzite’s coarse structure, influencing wear patterns. To measure and show this empirical observation, we must use a different microscopic scanning workflow to compare edges before and after the experiments, which was beyond this study’s scope that deals with edge wear (see Table 1). The results of physical properties (HLC hardness values) divides the materials into two groups, with quartzite emerging as distinctly softer. Logically, quartzite’s moderate hardness should predict high edge wear frequency. However, in Figure 9, we can observe the opposite. The relation between the proxies to estimate efficiency and Leeb values shows that quartzite is closer to cluster with flint as a highly efficient raw material than the fine grain or no grain raw materials. Although scatter plots indicate no clear relationship between hardness, PD, and Ew, statistical validation using larger datasets remains necessary.

Visual examination of all samples shows that the modification of the edge is caused mostly by micro-detachments as a result of the tribological interaction between three variables (active sample, contact material, and the outcome of the contact of the two samples’ micro-debris of rock and wood dust). Such micro-detachments occurred in all lithic raw materials at different stages. Micro-detachments have an important role when performing a task, in this case, bi-directional movement in pinewood, as the continuous detachment helps to refresh the edge and remove more of the target material on the following use. Flint samples, for example, showed no significant alteration. In this case, what likely happens is that the continuing use of the flint samples causes blunting through abrasion, finally resulting in a smoothed edge. However, sample FLT10−2 showed different mechanical behaviour. From 500 to 1000 strokes, a major detachment occurs and breaks the stage of dullness on the edge (S2). This detachment acts as an accidental mechanical “self-resharpening” action [27,84]. The recurrence of this mechanical phenomenon can dictate the performance of the edge, influencing the knapper to use different technological strategies or techniques (retouching or reshaping/rejuvenation) to manipulate/ “control” the edge according to the performed activity and a specific lithic raw material. Obsidian, for example, remained resilient in the first 250 strokes, besides some minor edge detachments. After the first stage, obsidian samples showed a wavy edge, almost identical to intentional retouching (S4). Our observations and values showed that this phenomenon increases the friction of this raw material, which is becoming highly reduced as more strokes are applied.

Our work shows that various lithic raw materials have a variable degree of efficiency and behave differently, likely due to intrinsic material properties. When translated to the archaeological record and human decision-making choices, these observations are crucial, as the outcome of selection, production, and use of lithic raw materials will ultimately focus on how the edge holds when performing a specific activity. As such, the higher proportion of one lithic raw material over the other in the archaeological record could be due to the degree of efficiency of lithic raw material in different activities.

Our experimental setting is a highly controlled environment built upon the uniformitarian law of mechanical fracture of lithic raw materials. The aim of this experimental work is not a direct link to the artefact outcome, rather, it is to assess and test the resources chosen and used by humans in the past. This was measured using the efficiency value of each rock type as a proxy. We built an experimental setup to show that if we use these two proxies (PD and Ew), we can achieve an efficiency value in this specific template for a sawing action but also for other actions that will measure the same proxies. However, the bridge with the archaeological assemblages can be done by the multiscale analysis of lithic technology, use-wear analysis, and now with a relation of rock types and efficiency values for each action.

In so, from this experimental setting two main observations can be drawn based on the quantitative data and visual documentation presented in this study. First having control over the edge morphology had to be of paramount importance for past hunter-gatherers, since, as shown in our experiments, this highly affects lithic performance. When there was limited availability of some raw materials or when access to some outcrops was hindered, past hunter-gatherers had to come up with technological solutions to mitigate such problems. Those solutions would target problems related to two extreme points of tool use but interlinked technologically, either to overcome the consecutive damage of the tool edge or to overcome the rather quicker dullness of the edge. The identification of such solutions has been gradually introduced in some archaeological case studies, such as the work of Frison [85], as part of a broad spectrum of flakes classified as retouching and sharpening flakes. These types of technological solutions have one aspect in common, which is to overcome the problems mentioned above to extend the use of the raw material being exploited. In this scenario, the life-extension of the exploited raw material can be accomplished either by the application of retouch to manipulate/control the tool edge or by specific technological removals (i.e., large sharpening flakes [67]. Morales and Vergès [86] presented a wide set of examples based on this topic, specifically resharpening flakes that clean and maintain the edge to preserve the mass of the tool. These observations were also made in ethnographic studies. For example, in Ethiopian communities that use lithic scrapers for the treatment and processing of hide, it was reported that the resharpening of such tools acts as a technical behaviour to rejuvenate the edges when they become dull [89]. In the case of the Ngaanyatjarra tulas, evidence of resharpening was documented in wood scraping activities [90]. Also, among the Tehuelche scrapers, the resharpening of two raw materials (glass and chalcedony) was done regularly [91]. These findings parallel our observations of flint and obsidian samples requiring regular retouching.

Such data enrich theoretical aspects of lithic studies, particularly Binford’s [92] curation concept, emphasising raw material utility maximisation through maintenance and recycling linked with procurement strategies. Curation is seen as a relationship between variables in the same way we predicted efficiency. Lithic tools, according to their size, design, and properties, have a finite amount of value/utility [93,94]. As such, curation is the degree of use or utility extracted, expressed as a relationship between the maximum utility a tool starts with and how much of that utility is realised before it is discarded [95]. When dealing with curation through utility, as proposed by Shott [94], we deal with the functional value of the tool. By testing the performance of stone tools using the efficiency equation proposed in this work, it is possible to understand the functional value of the different tools and raw materials.

**Fig 14:**
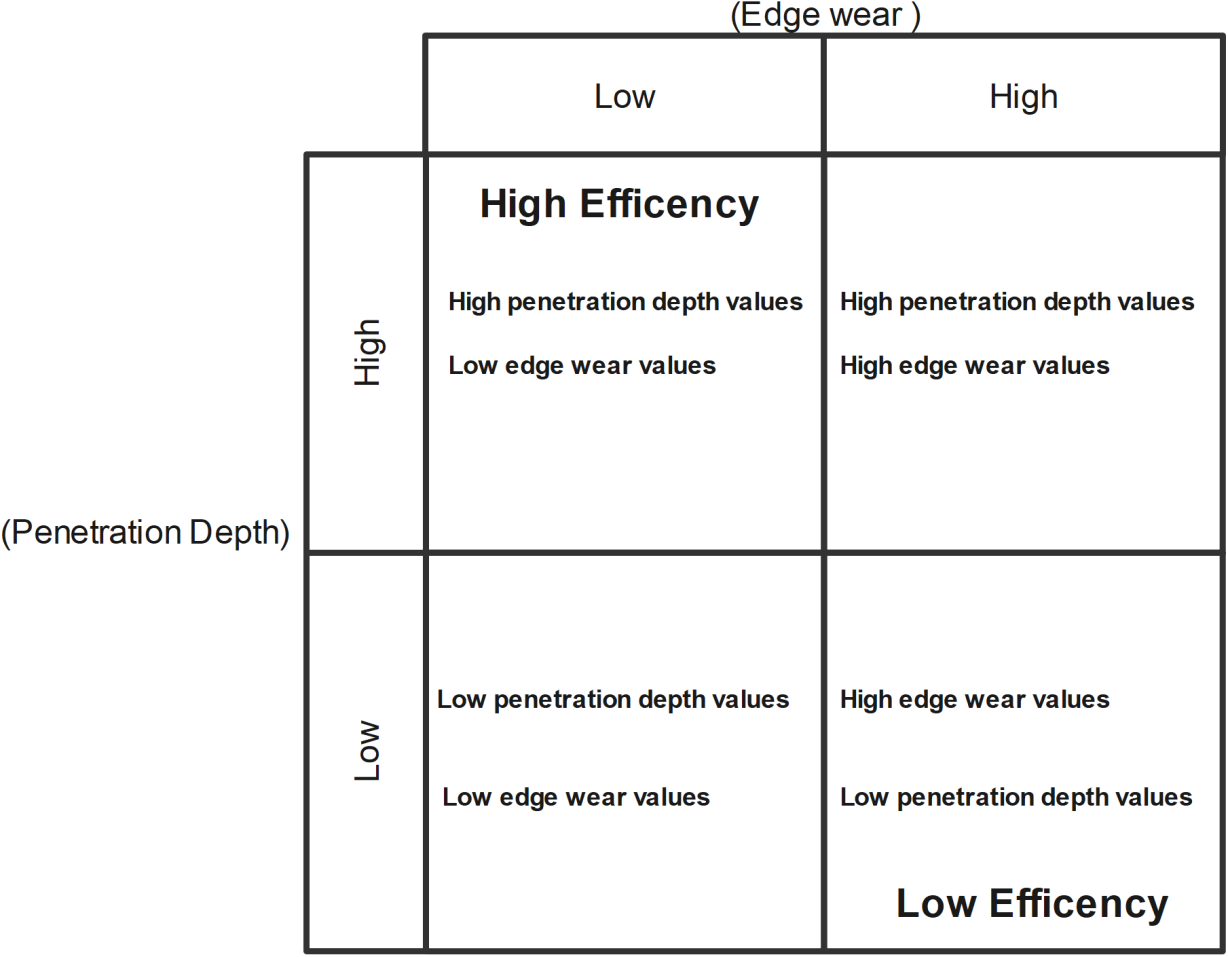
Efficiency matrix categorising materials based on penetration depth and edge wear. High-efficiency materials combine high penetration with lower edge wear, and Low-efficiency raw materials combine low penetration and high edge wear.

Second, interlinked with the first, our approach contributes methodologically to Human Behavioural Ecology (HBE), emphasising optimality principles in technological decision-making [96]. Despite focusing on one activity (sawing), the quantitative classification we propose, based on PD versus Ew serves as a guideline for efficiency evaluation across diverse activities (motions) and contact materials. The theoretical diagram we proposed in Figure 14 is the result of a qualitative classification of raw materials’ performance driven by a quantitative set of selected key proxies. The increase of controlled experiments applied to decision-making that use the same proxies can now build mechanical behaviour values and use them to make inferences about the past technological systems (procurement/production/use of stone tools). In this line of thought, we propose that this methodology would contribute to a better understanding of models that rely on the marginal value theorem (MVT), which sees stone tools as patches [97]. MVT delves into predictions to understand foraging behaviour. This model assumes that foragers should give up/leave a patch when the instantaneous returns drop below the average return rates for other patches.

Lithic raw materials can be classified as patches of utility. Kunh and Miller [45] stated that *“this utility comes from the fact that artefacts provide a mechanical advantage that makes certain tasks possible or makes them more time or energy efficient. In other words, although artefacts do not directly supply energy or nutrients to users, by making work more efficient, a usable implement or weapon has the potential to produce a net energy gain for a tool user”*. Also, artefact utility is finite, and its utility will decline over time as the artefact is used, wears out, and approaches a point of failure. The remaining questions are about how long people should use lithic raw material and whether we can predict the utility of each raw material to assume that past hunter-gathers could give up/leave a patch.

Here, we suggest that our work can contribute specifically to the procurement and use of past lithic technologies. Using the optimal foraging tradition’s cost/benefit logic, we propose that the efficiency values can be used in models that explore those same activities and estimate rates of lithic raw materials as patches.

## Conclusion

This study provides a quantitative analysis of the efficiency of four distinct lithic raw materials: flint, obsidian, quartzite, and dacite, through controlled experiments. Our findings highlight significant differences in the mechanical performance of these materials, specifically in terms of penetration depth (effectiveness) and edge wear (durability). Flint demonstrated the highest overall efficiency, characterised by consistent performance with increasing penetration depth and moderate edge wear. While showing initial similarities to flint, dacite exhibited an efficiency plateau during the later stages of the experiment. Quartzite, despite its initial high edge fragmentation and wear, showed improved performance over time, stabilising its edge and increasing penetration depth. Obsidian, although highly brittle and prone to edge wear, maintained its effectiveness, albeit with higher fragmentation rates.

Using hardness as the main proxy to test the dichotomy of the fine versus coarse grain classification showed us that the regular classification of grain size is insufficient when dealing with the complexity of lithic raw materials. For example, we showed that quartzite, aside from flint, is efficient in this bi-directional setup. This could explain why it appears in the archaeological record across time and space, even when humans occupy regions that are rich in fine-grain raw materials. These results suggest that the mechanical properties of lithic raw materials significantly influenced the raw material selection and technological choices of past hunter-gatherer communities. The efficiency of these materials would have been a critical factor in their selection for tool production and use, impacting the procurement, production, and maintenance strategies of prehistoric toolmakers. By quantifying the efficiency of different lithic raw materials, this study contributes to our understanding of human decision-making processes and the technological adaptations that shaped the evolutionary trajectory of stone tool technologies with relevance for their earliest stages.

It should be stated that this study should not be seen as a generalisation of all varieties of represented lithic raw material or performed activities. This work aimed to show that efficiency values can be extracted from lithic raw materials that were used in the past. Those values are different from raw material to raw material, but they are expected to change according to the activity/ motion performed.

These controlled experiments represent a first step toward building a comparative framework to better understand prehistoric tool use and behavioural variability.

## Supporting information

SOM1 Imaging equipment, acquisition settings. (ZIP)

SOM2 GOM Inspect and CloudCompare workflow for 3D mesh processing, alignment and computing. (ZIP)

SOM3 Folder with high-speed camera videos of the experiments. (DOCX)

SOM4 Folder containing Leeb rebound hardness reports for all raw materials. (ZIP)

SOM5 Folder with videos of the lab experimentation. (DOCX)

SOM6 Folder with digital images of the edge wear on all raw material samples. (ZIP)

SOM7 Folder with all the R scripts for data processing and analysis. (ZIP)
