# Supplementary material for: The dichotomy of human decision-making: An experimental assessment of stone tool efficiency
Source: PLoS One. 2025 Jul 18;20(7):e0327215. doi: 10.1371/journal.pone.0327215 (PMC12273975; doi:10.1371/journal.pone.0327215)
Supplement: SOM1 — (ZIP) [file pone.0327215.s001.zip › SOM_1_Imaging_equipment_acquisition_settings/SOM1_Acquisition settings.pdf]

Nora et al. The Dichotomy of Human Decision-Making: from tThe Impact of Lithic Raw Material Properties on Stone Tool Efficiency.

SOM1 – Acquisition settings

Imaging technique	Equipment	Objective	FOV	Resolution
Photography	Nikon DSLR D160	Nikon AF-S VR Micro-Nikkor 105 mm f/2.8G IF-ED		
3D scanner	HP Pro S3 David SLS-3		Up to 120 mm	up to 0.06 mm
3D digital microscope	ZEISS Smartzoom 5	PlanApo 1.6×/0.1		
