# Supplementary material for: The dichotomy of human decision-making: An experimental assessment of stone tool efficiency
Source: PLoS One. 2025 Jul 18;20(7):e0327215. doi: 10.1371/journal.pone.0327215 (PMC12273975; doi:10.1371/journal.pone.0327215)
Supplement: SOM2 — (ZIP) [file pone.0327215.s002.zip › SOM_2_GOM_Inspect_Cloud_Compare__3D_mesh_processing_alignment_computing/CloudCompare workflow.pdf]

CloudCompare workflow:

1. **Import** the two .stl files (before and after
2. **Align** 3D models
    a. **Select the two meshes**.
    b. Registration **and Match Bounding-box centers**
    c. Registration and **Align** (points pair picking)
    d. Registration and **Fine registration** (ICP)
3. **Compare** the two meshes[1]
    a. **Select the two** meshes
    b. Compute **Cloud/Mesh distance**
    c. Compute
    d. Select only the "**registered**" mesh
    e. In **Properties/SF display params/Display ranges**
        i. Set displayed values to 0.2 mm (3D Scanner accuracy and baby powder layer)
        ii. Make the Color Scale visible
        iii. In the Parameters bar **unselect "show NaN/ out of range values in grey"**
4. Get **comparison** data
    a. Select only the "registered" mesh
    b. Show Histogram
    c. Export Histogram to a .CSV file
    d. Export Histogram to image to .PNG

[1] Combinations: Stage 1(0-125 cycles); Stage 2 (126-250 cycles), Stage 3 (251-500 cycles) and Stage 1 with Stage 3 (0-500 cycles)

CloudCompare Version 2.12 alpha [Windows 64-bit] *Complied with MSVC 1916 and 5.15.2*
License: GNU GPL (General Public Licence).
