# Supplementary material for: The dichotomy of human decision-making: An experimental assessment of stone tool efficiency
Source: PLoS One. 2025 Jul 18;20(7):e0327215. doi: 10.1371/journal.pone.0327215 (PMC12273975; doi:10.1371/journal.pone.0327215)
Supplement: SOM2 — (ZIP) [file pone.0327215.s002.zip › SOM_2_GOM_Inspect_Cloud_Compare__3D_mesh_processing_alignment_computing/GOM Inspect Workflow.pdf]

Nora et al. 2024 - The Dichotomy of Human Decision-Making: The Impact of Lithic Raw Material Properties on Stone Tool Efficiency.

GOM Inspect Workflow

Mesh Treatment

1. **Import** the stage 0 .stl file (alternative: ply) import STL as target element type: mesh → ok

2. **Eliminate Mesh Errors**
   a. RMC (right mouse click) **Select all points of Element**
   b. **Operations → Mesh → Other → Eliminate Mesh Errors → Apply**

3. **Align 3D model**
   a. RMC on the Left back of the screen X, Y, Z icon to establish Matrix
   b. RMC **Select all points of Element**
   c. **Operations → Alignment → Manual Alignment →** Set Matrix (Settings[1])
   d. Change Rotation values until your object is aligned
   e. Change Translation to set your object to the 0/0/0 (zero) point[2] → **Ok**

4. **Cut 3D model**
   a. RMC → **Select/Deselect Trough Surface** (Ctrl+Shift+Space) → Close the select area LMC
   b. **Delete** select 3D area (Ctrl+Del)

5. **Export Mesh** as a .stl file. (File → Export → .stl).

6. **Close Project.**

---

[1] Coordinate System: Global coordinate system

Part choice: Part.

[2] Note: Check always different position by RMC on the left back X / Y / Z Icon.

GOM Software 2020 (2020 Hotfix 5, Rev. 139680, Build 2021/06/18)

Aligned Cut

1. **Cut 3D Models aligned**
   a. **Import** the stage 0 (zero) .stl file **aligned cut** (add Part)
   b. RMC **Select all points of Element → Operations → CAD → Actual Mesh to CAD**
2. **Import** mesh stg250_rawdata .stl file (second 3D model)
   a. **Operations → Alignment → Initial Alignment → Prealignment**[3] (At this stage both 3D models are overlap).

   b. **Cut the mesh** at the same line as the stage 0 (zero) → RMC → **Select/Deselect Trough Surface** (Ctrl+Shift+Space) → Close the select area LMC
7. **Export Mesh** as a .stl file. (File → Export → .stl)
8. **Repeat the aligned cut for the others 3D's models.**

[3] Search Time: Long

GOM Software 2020 (2020 Hotfix 5, Rev. 139680, Build 2021/06/18)
