# Supplementary material for: The dichotomy of human decision-making: An experimental assessment of stone tool efficiency
Source: PLoS One. 2025 Jul 18;20(7):e0327215. doi: 10.1371/journal.pone.0327215 (PMC12273975; doi:10.1371/journal.pone.0327215)
Supplement: SOM4 — (ZIP) [file pone.0327215.s004.zip › SOM_4_Leeb_reports/FLT10-2.pdf]

Equotip measurement report

**Measurement series file name**	FLT10-2
**Measurement mean hardness**	965.5 HLC

Measurement bar graph

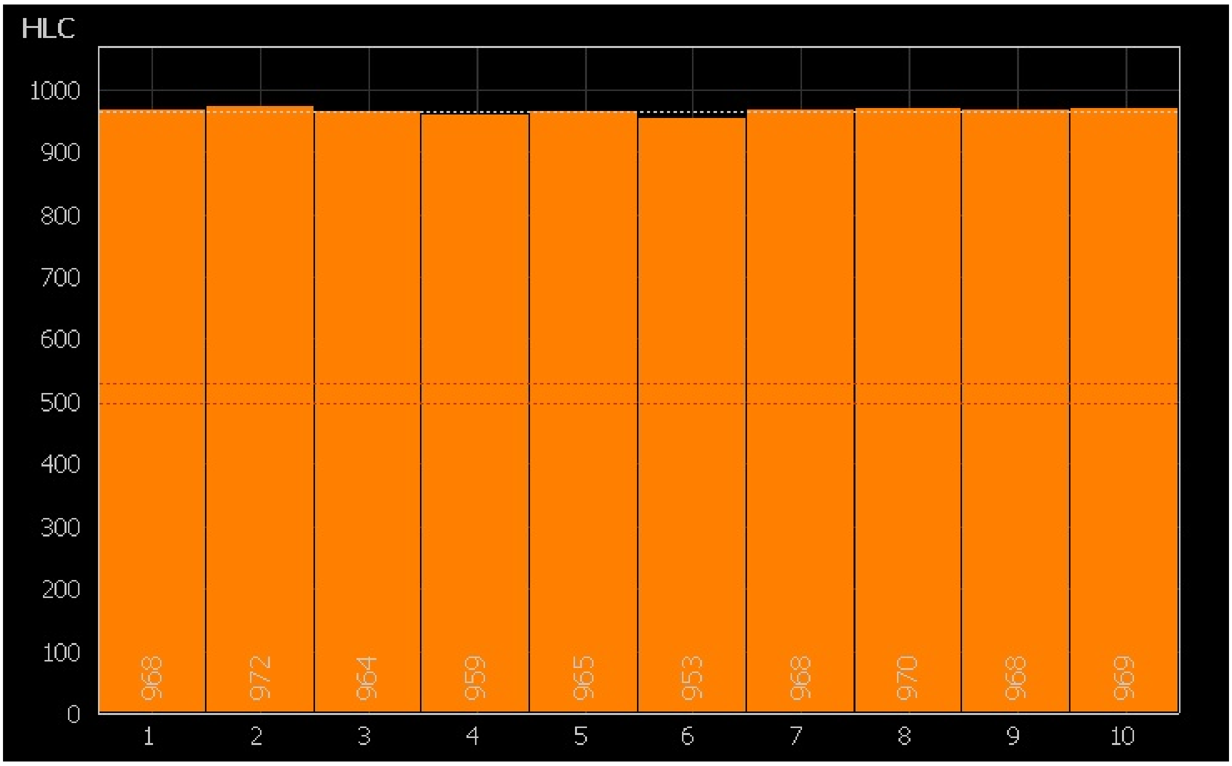

Sample Ids

Location
Company
Sample material
Sample ID
Lot

Device Info

Probe type	Equotip Leeb Impact Device C
Probe Serial Number	IC51-004-0185
Product Name	Equotip®
Product Version	550
Device Serial Number	UP01-003-1680
Hardware Revision	C1
Operating System Software	2.2.2
Application Software Version	2.7.0

Settings

Probe type	Equotip Leeb Impact Device C
Material	Flint (Custom Material)
Primary scale	HLC

	Conversion standard	Default
	Limits	High: 528 , Low: 498
**Statistics**		
	n	10
	$\bar{x}$	965.5 HLC
	σ	5.5 HLC
	Minimum / Maximum	953 / 972 HLC
	Range	19 HLC
**Operator**		Walter
