# Supplementary material for: The dichotomy of human decision-making: An experimental assessment of stone tool efficiency
Source: PLoS One. 2025 Jul 18;20(7):e0327215. doi: 10.1371/journal.pone.0327215 (PMC12273975; doi:10.1371/journal.pone.0327215)
Supplement: SOM5 — (DOCX) [file pone.0327215.s005.docx]

S5

For the videos as supplementary information, please follow this DOI:

Nora, D. (2024). The Dichotomy of Human Decision-Making: The Impact of Lithic Raw Material Properties on Stone Tool Efficiency. Zenodo. <https://doi.org/10.5281/zenodo.14012778>
