# Supplementary figures and images for: The dichotomy of human decision-making: An experimental assessment of stone tool efficiency
Source: PLoS One. 2025 Jul 18;20(7):e0327215. doi: 10.1371/journal.pone.0327215 (PMC12273975; doi:10.1371/journal.pone.0327215)

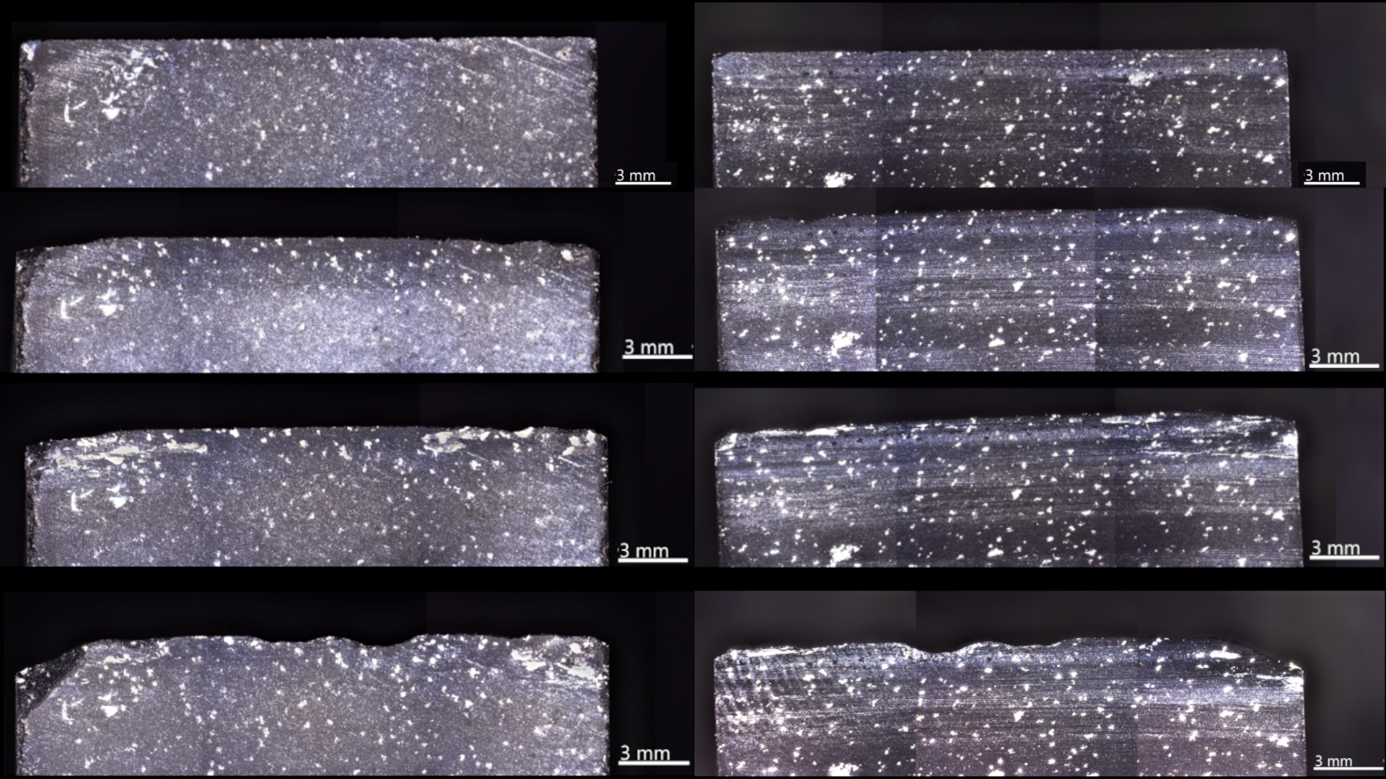

Supplement: SOM6 — (ZIP) [file pone.0327215.s006.zip › SOM_6_Zeiss_Smart_Zoom/ZEISS Smart zoom DAC3-2_left_back_view_Right_front_view_top_ to_bottom_cycle_0_125_250_500.png]

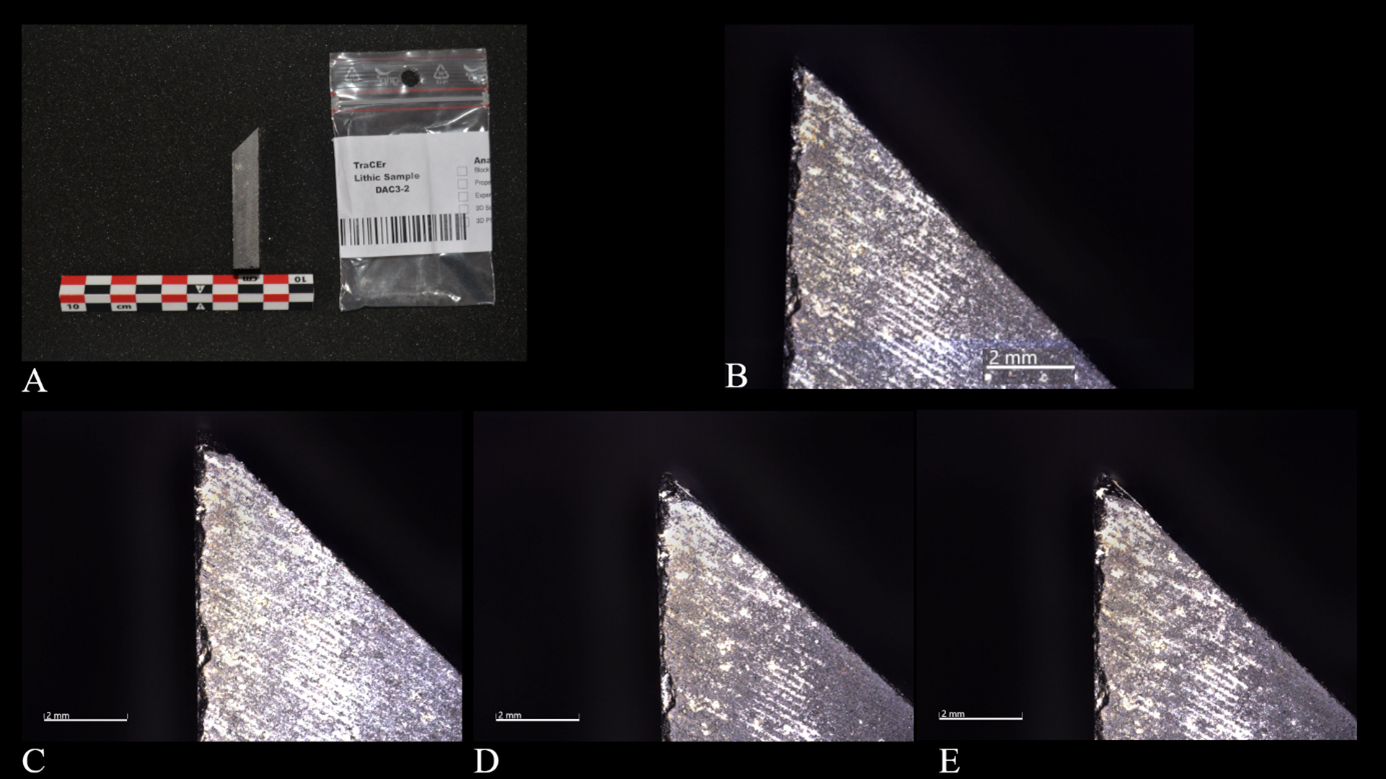

Supplement: SOM6 — (ZIP) [file pone.0327215.s006.zip › SOM_6_Zeiss_Smart_Zoom/ZEISS Smart zoom DAC3-2_Profile view, A – Sample_ ID; B – cycle 0; C – cycle 125; D - cycle 250; E – cycle 500.png]

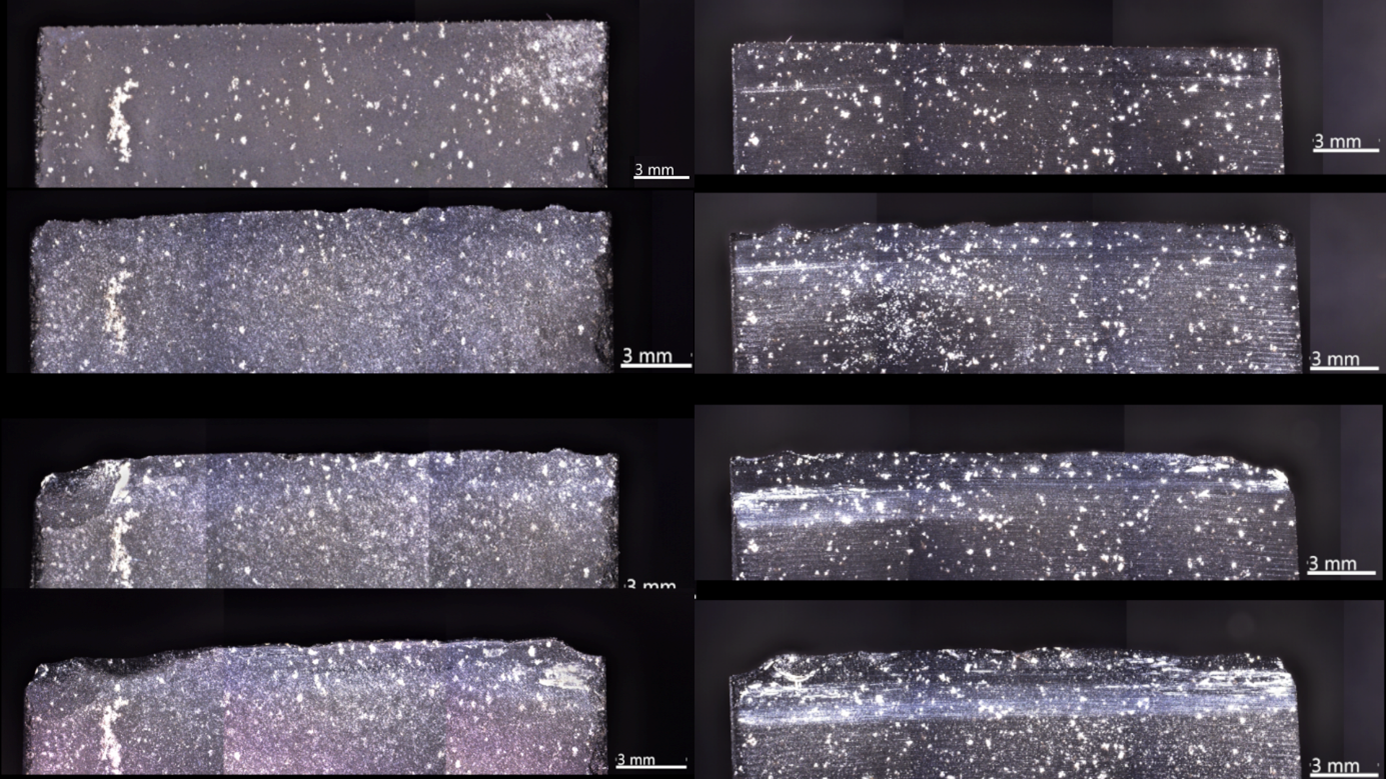

Supplement: SOM6 — (ZIP) [file pone.0327215.s006.zip › SOM_6_Zeiss_Smart_Zoom/ZEISS Smart zoom DAC3-4_left_back_view_Right_front_view_top_ to_bottom_cycle_0_125_250_500.png]

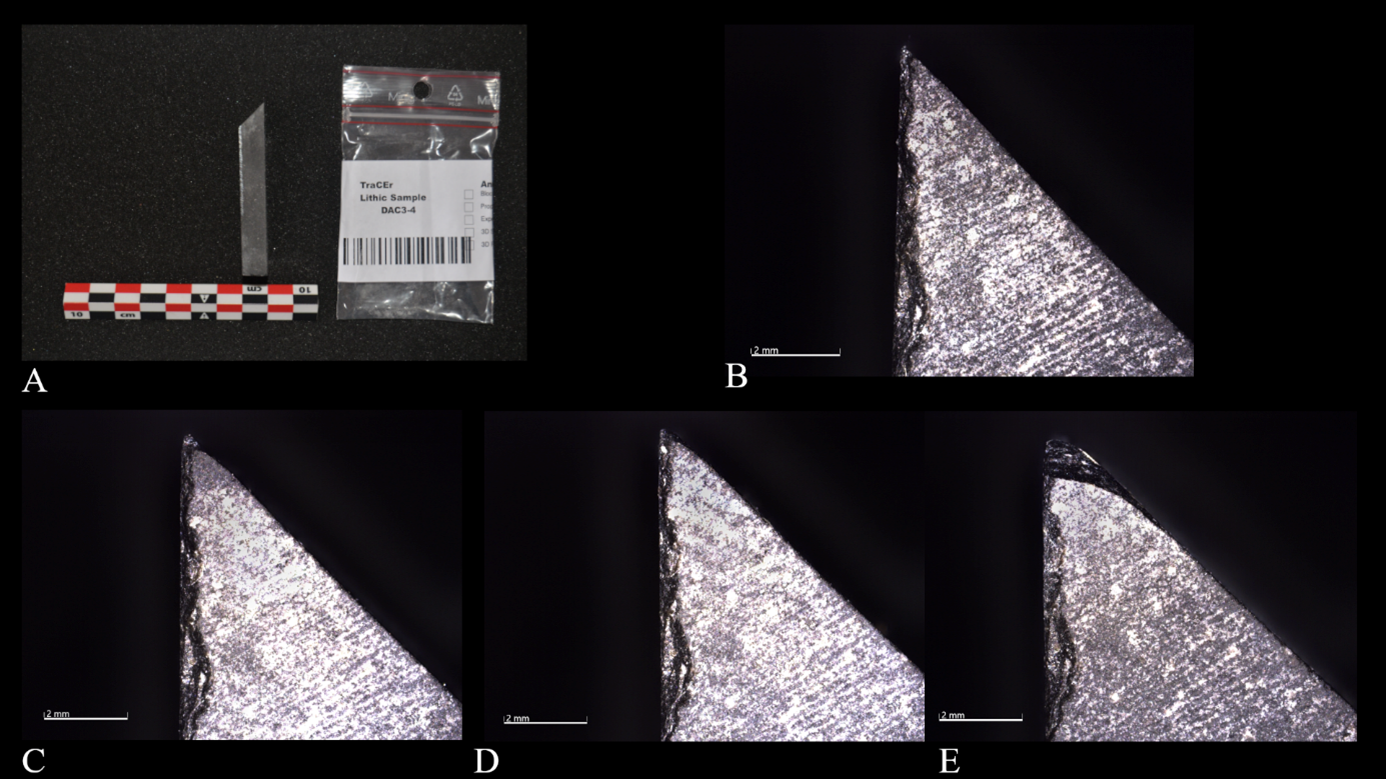

Supplement: SOM6 — (ZIP) [file pone.0327215.s006.zip › SOM_6_Zeiss_Smart_Zoom/ZEISS Smart zoom DAC3-4_Profile view, A – Sample ID; B – cycle 0; C – cycle 125; D - cycle 250; E – Cycle 500.png]

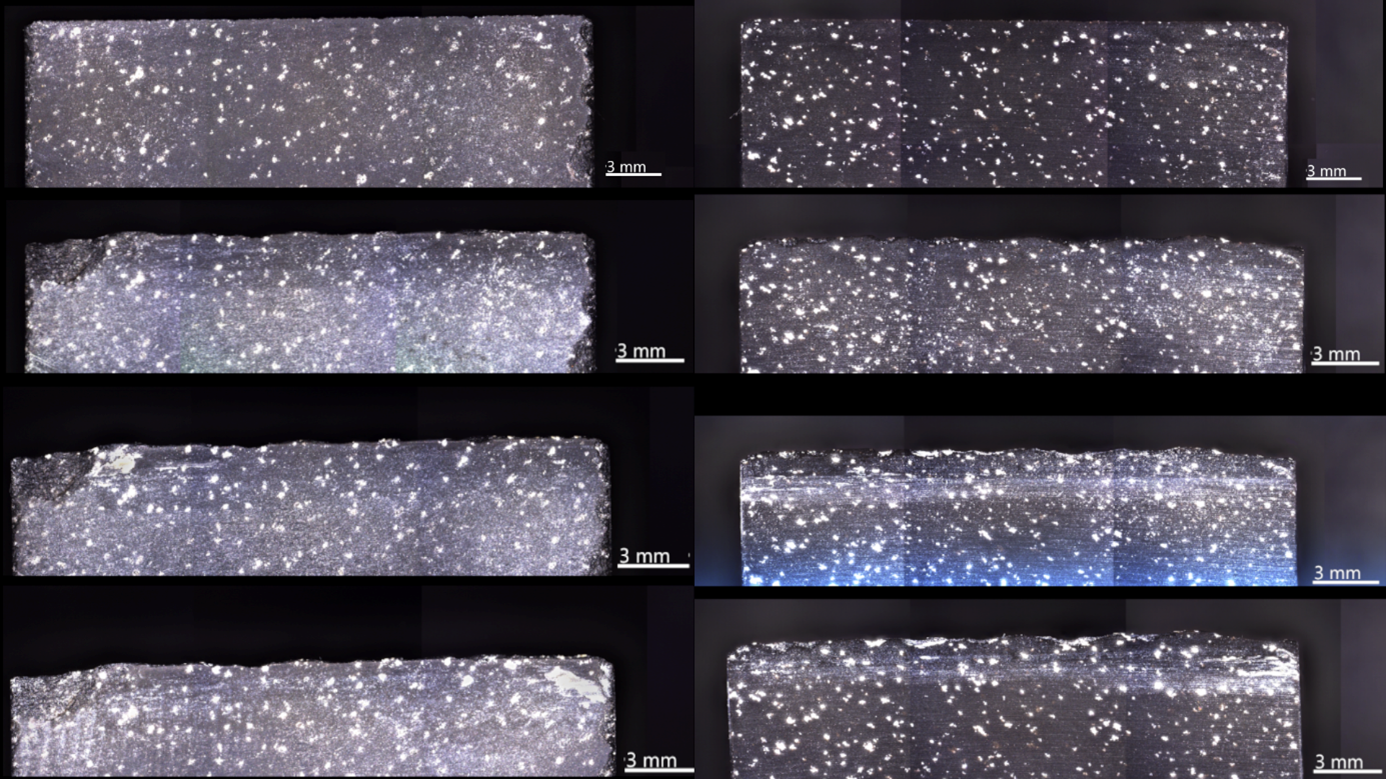

Supplement: SOM6 — (ZIP) [file pone.0327215.s006.zip › SOM_6_Zeiss_Smart_Zoom/ZEISS Smart zoom DAC3-6_left_back_view_Right_front_view_top_ to_bottom_cycle_0_125_250_500.png]

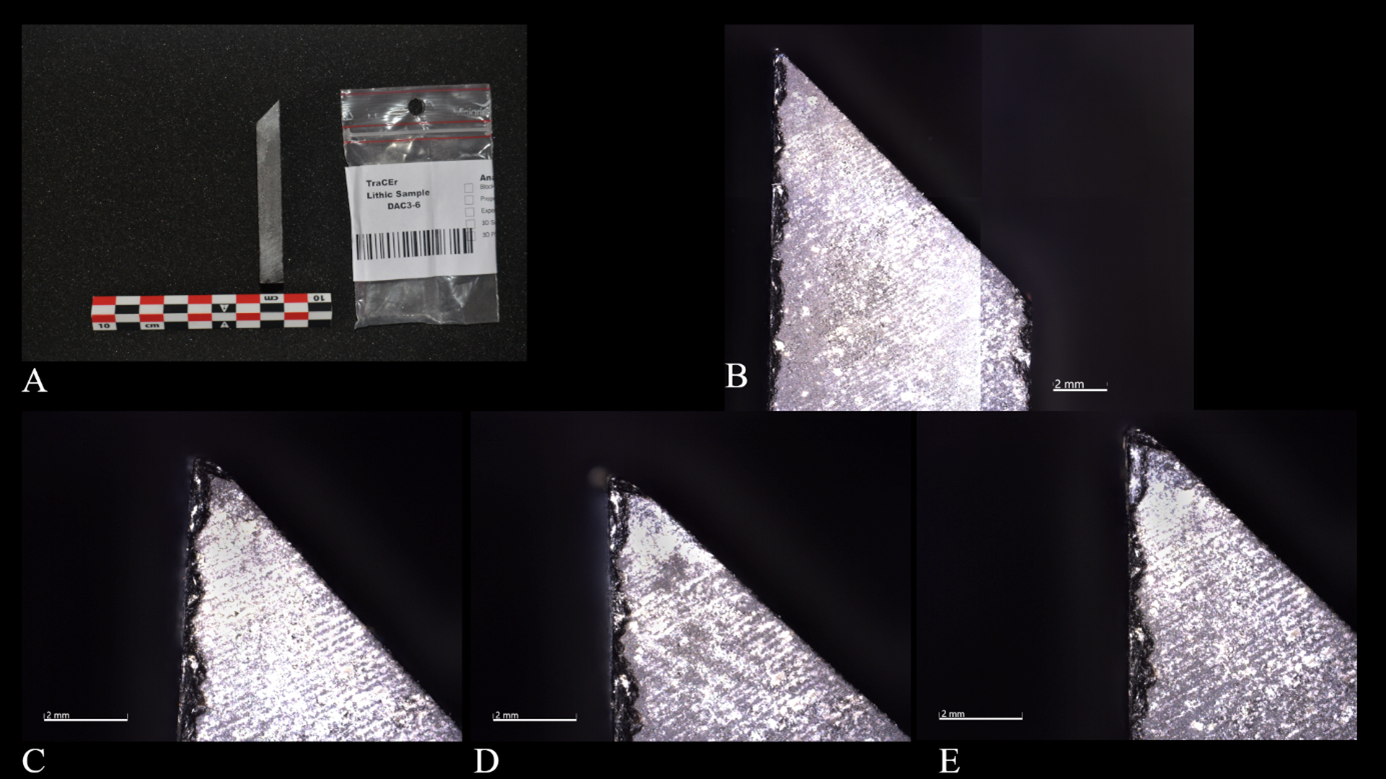

Supplement: SOM6 — (ZIP) [file pone.0327215.s006.zip › SOM_6_Zeiss_Smart_Zoom/ZEISS Smart zoom DAC3-6_Profile view, A – Sample ID; B – cycle 0; C – cycle 125; D - cycle 250; E – cycle 500.png]

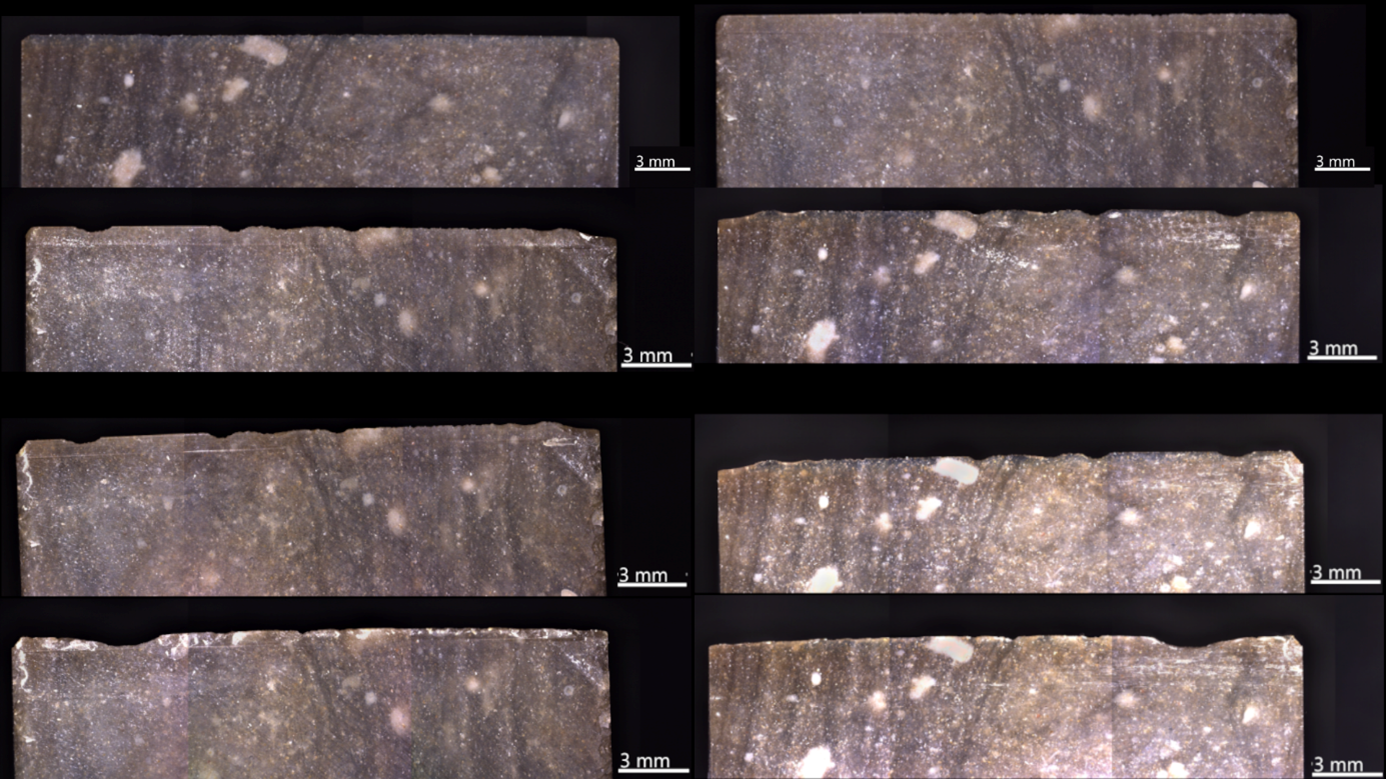

Supplement: SOM6 — (ZIP) [file pone.0327215.s006.zip › SOM_6_Zeiss_Smart_Zoom/ZEISS Smart zoom FLT10-2_left_back_view_Right_front_view_top_ to_bottom_cycle_0_125_250_500.png]

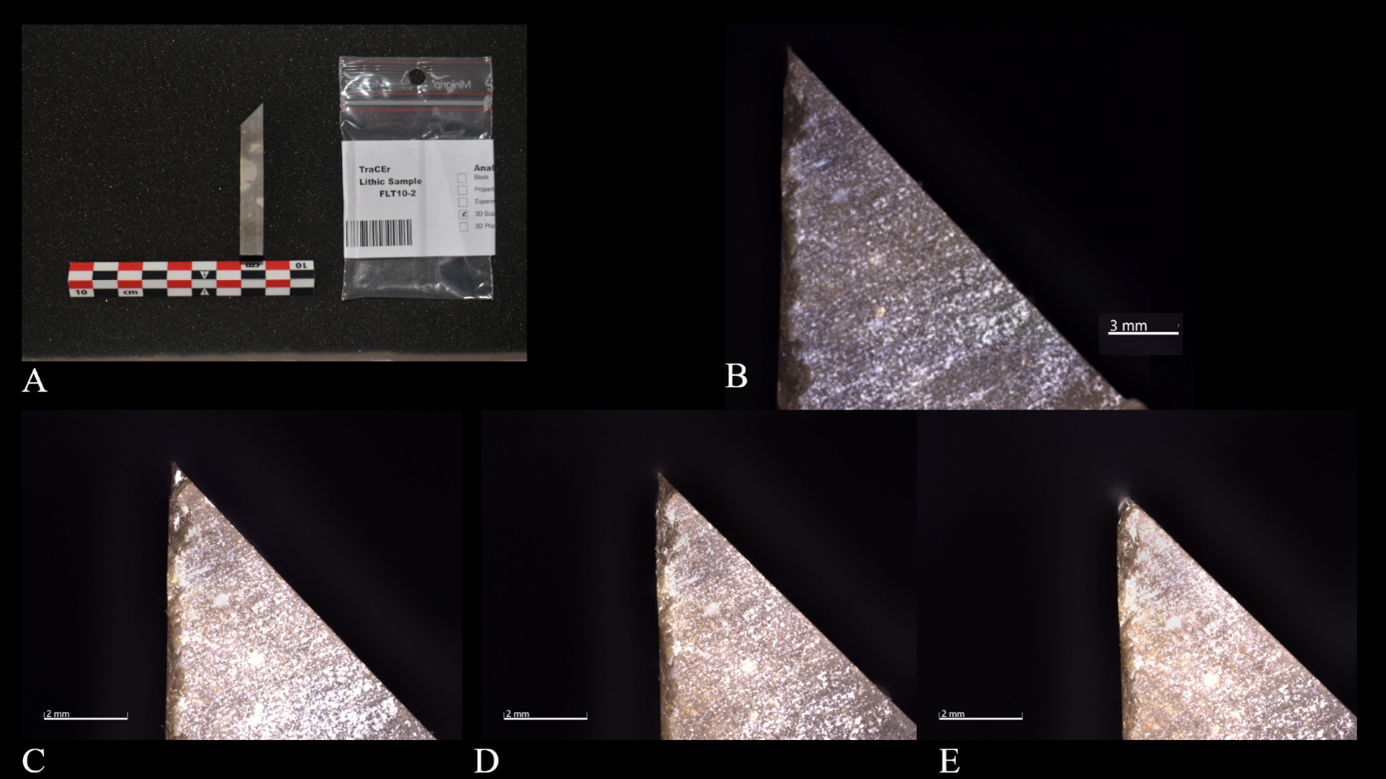

Supplement: SOM6 — (ZIP) [file pone.0327215.s006.zip › SOM_6_Zeiss_Smart_Zoom/ZEISS Smart zoom FLT10-2_Profile view, A – Sample ID; B –cycle 0; C – cycle 125; D - cycle 250; E – cycle 500.png]

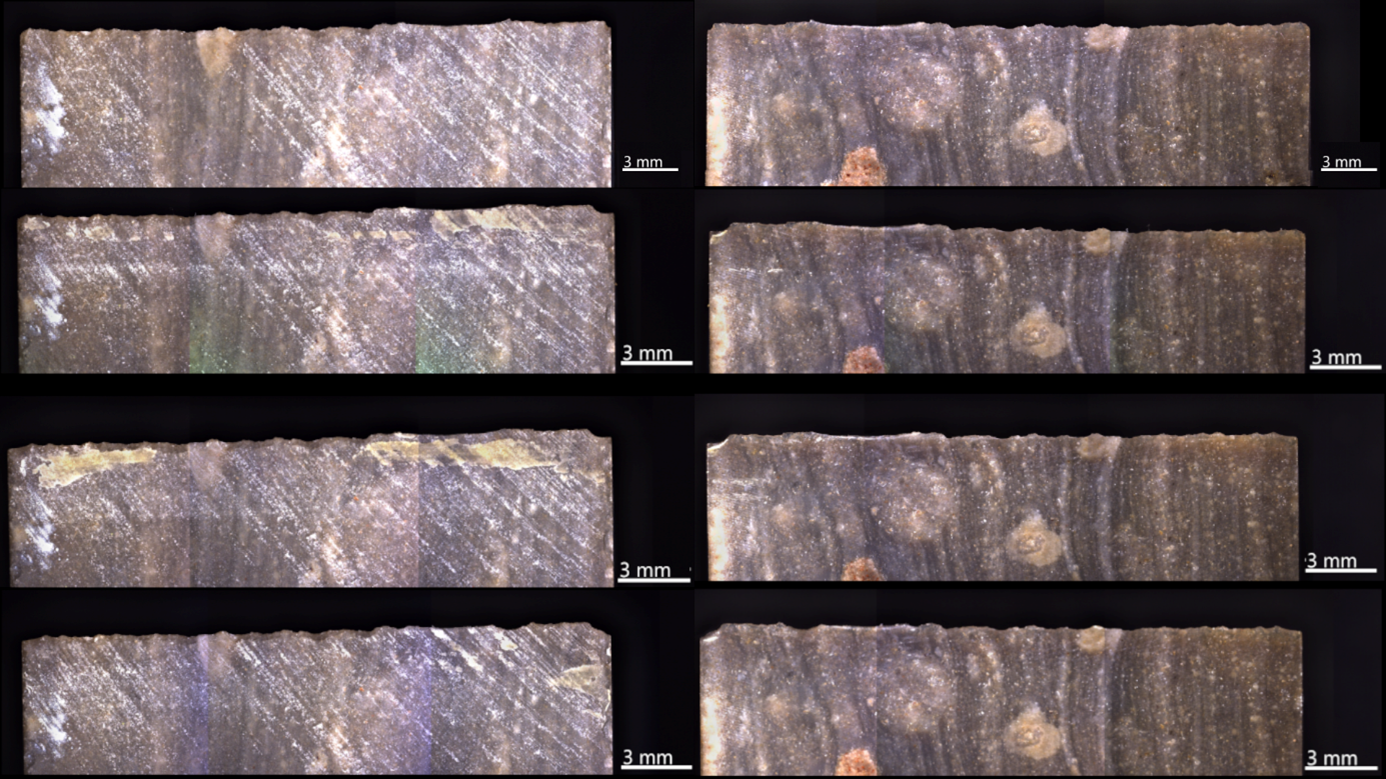

Supplement: SOM6 — (ZIP) [file pone.0327215.s006.zip › SOM_6_Zeiss_Smart_Zoom/ZEISS Smart zoom FLT10-5_left_back_view_Right_front_view_top_ to_bottom_cycle_0_125_250_500.png]

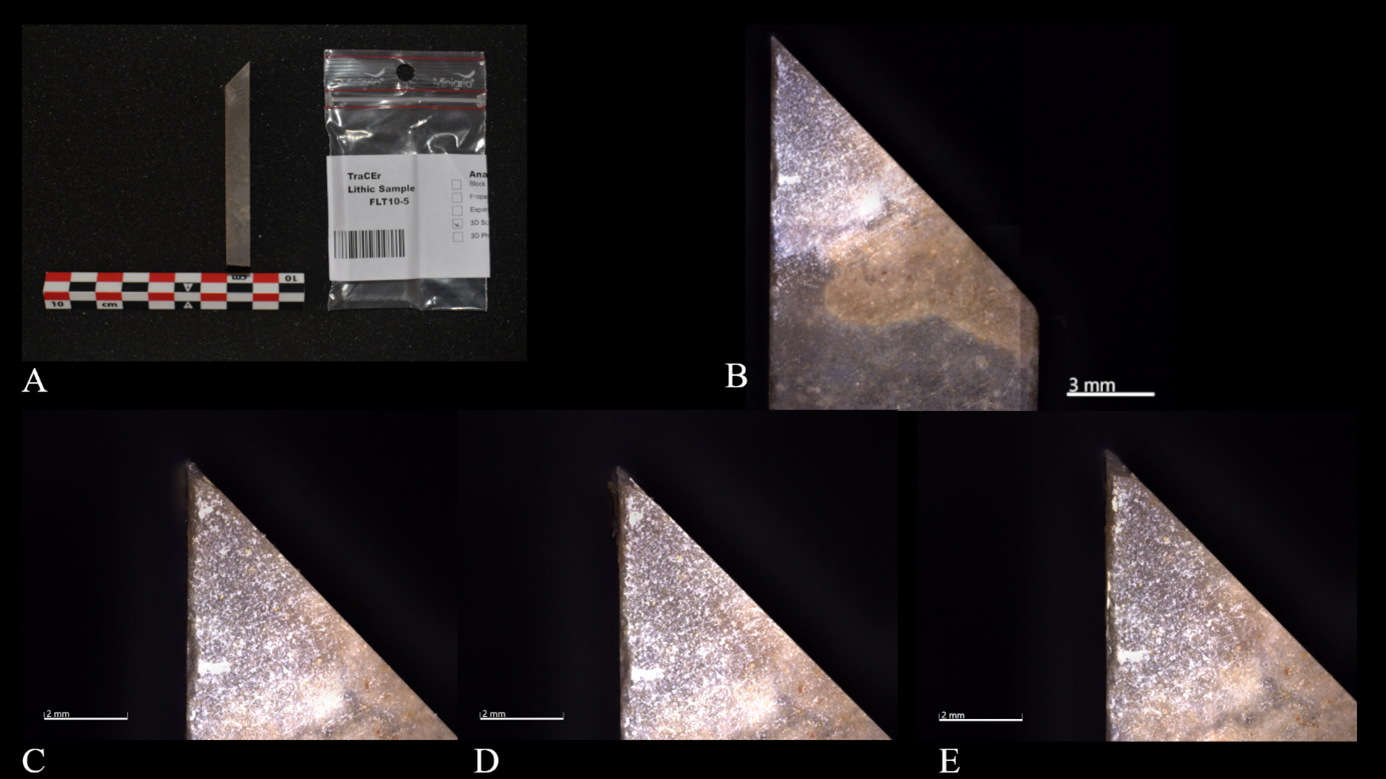

Supplement: SOM6 — (ZIP) [file pone.0327215.s006.zip › SOM_6_Zeiss_Smart_Zoom/ZEISS Smart zoom FLT10-5_Profile view, A – Sample ID; B – cycle 0; C – cycle 125; D - cycle 250; E – cycle 500.png]

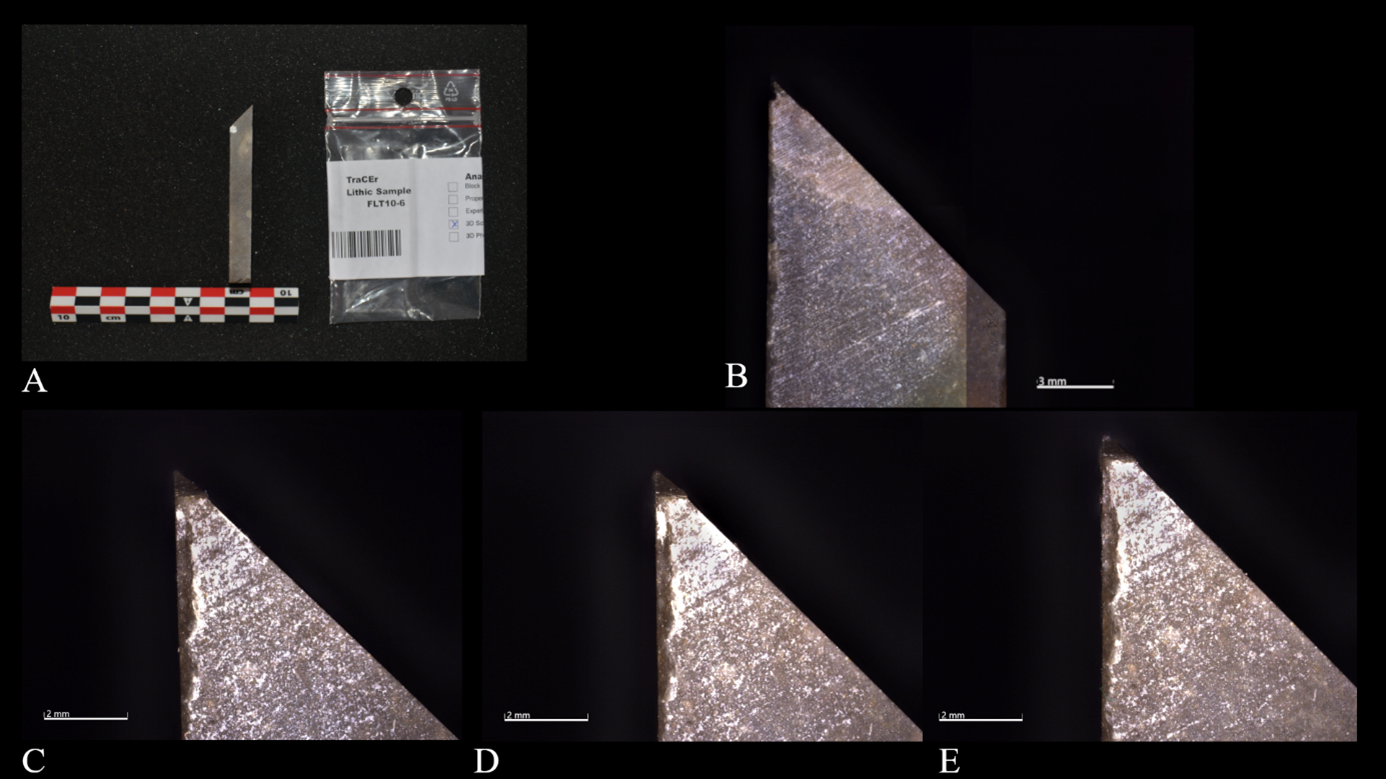

Supplement: SOM6 — (ZIP) [file pone.0327215.s006.zip › SOM_6_Zeiss_Smart_Zoom/ZEISS Smart zoom FLT10-6_ Profile view, A – Sample ID; B –cycle 0; C – cycle 125; D - cycle 250; E - cycle 500.png]

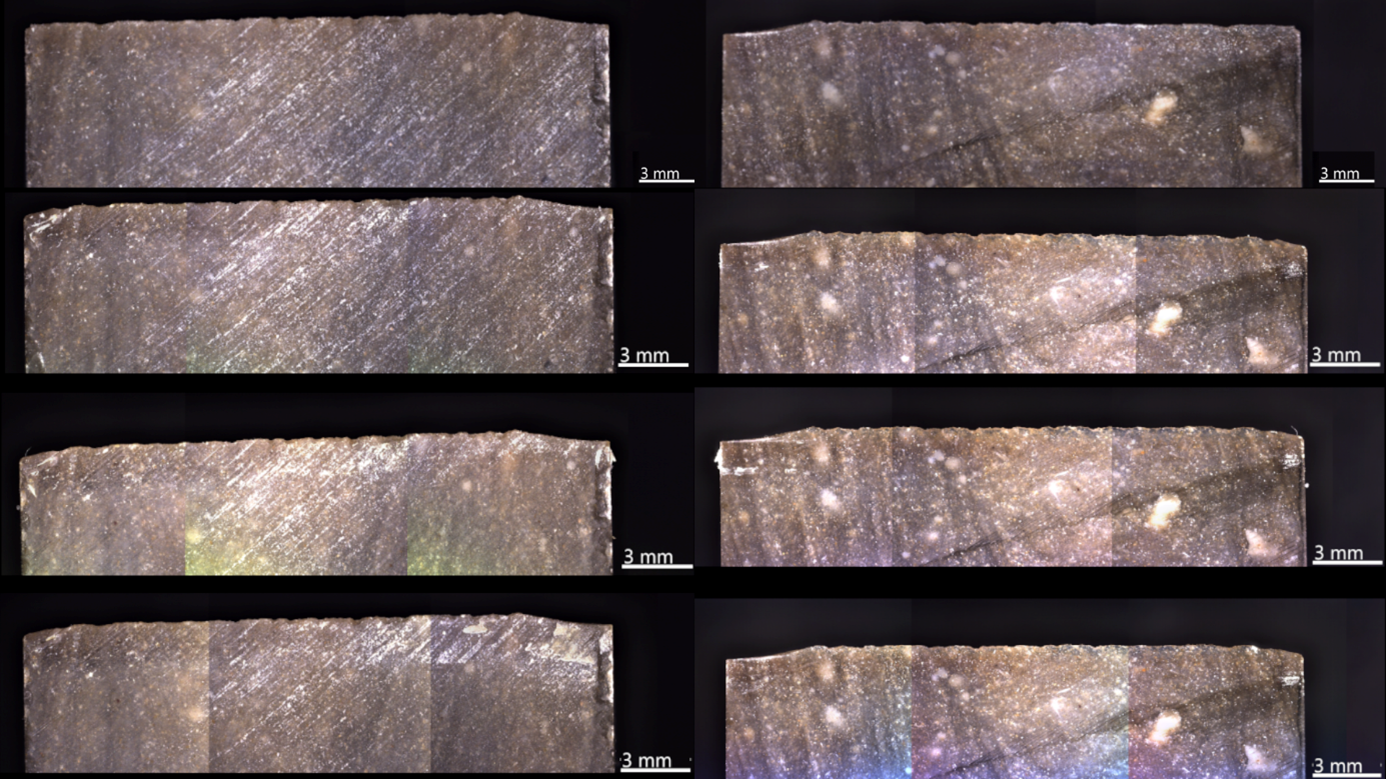

Supplement: SOM6 — (ZIP) [file pone.0327215.s006.zip › SOM_6_Zeiss_Smart_Zoom/ZEISS Smart zoom FLT10-6_left_back_view_Right_front_view_top_ to_bottom_cycle_0_125_250_500.png]

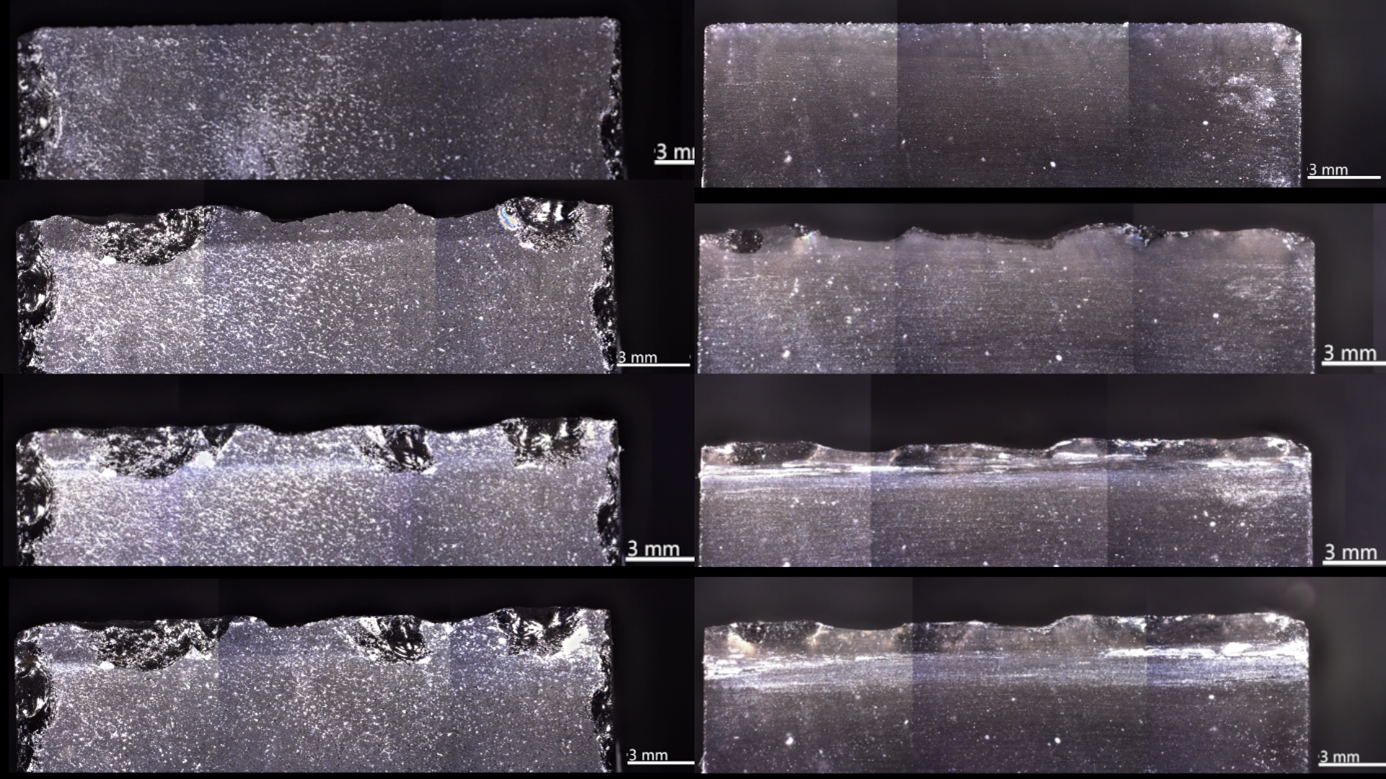

Supplement: SOM6 — (ZIP) [file pone.0327215.s006.zip › SOM_6_Zeiss_Smart_Zoom/ZEISS Smart zoom OBS4-4_left_back_view_Right_front_view_top_ to_bottom_cycle_0_125_250_500.png]

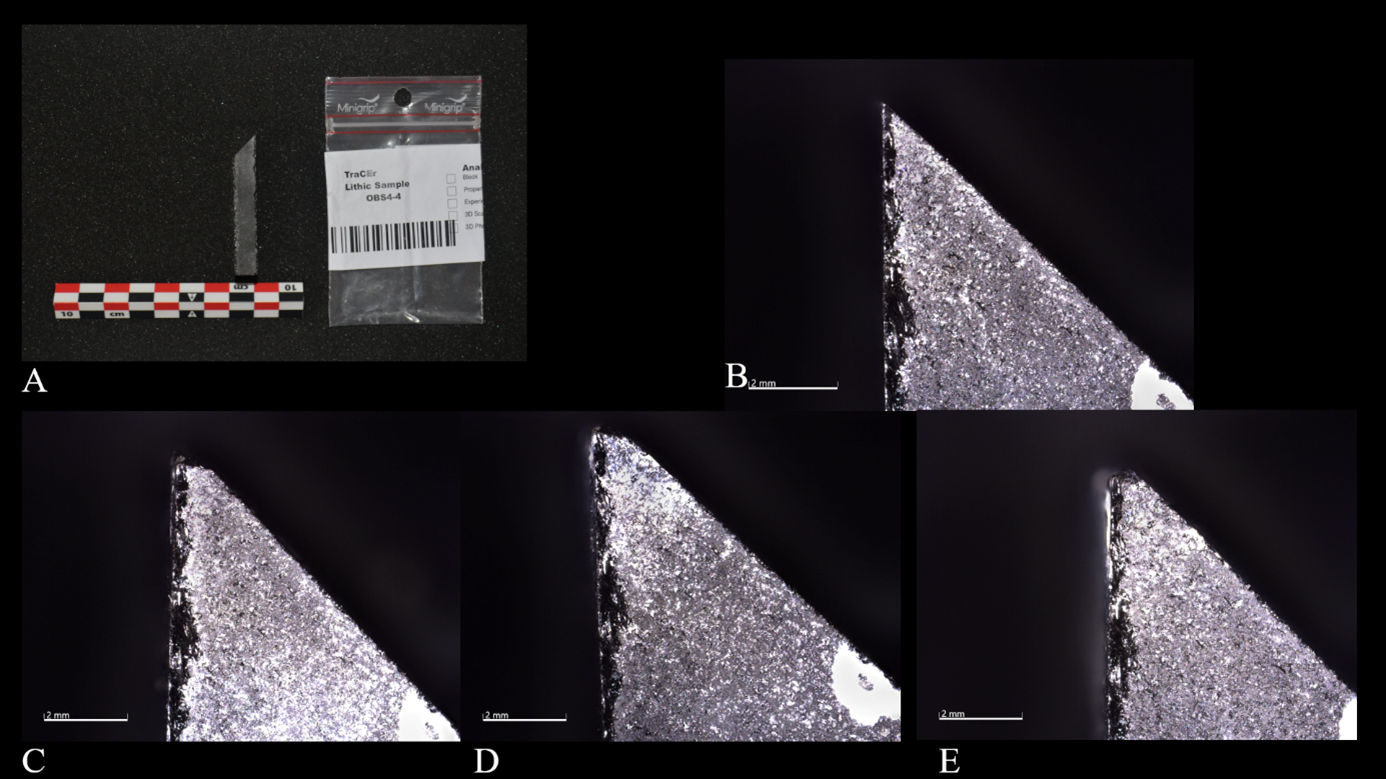

Supplement: SOM6 — (ZIP) [file pone.0327215.s006.zip › SOM_6_Zeiss_Smart_Zoom/ZEISS Smart zoom OBS4-4_Profile view, A – Sample ID; B – cycle 0; C – cycle 125; D - cycle 250; E – cycle 500..png]

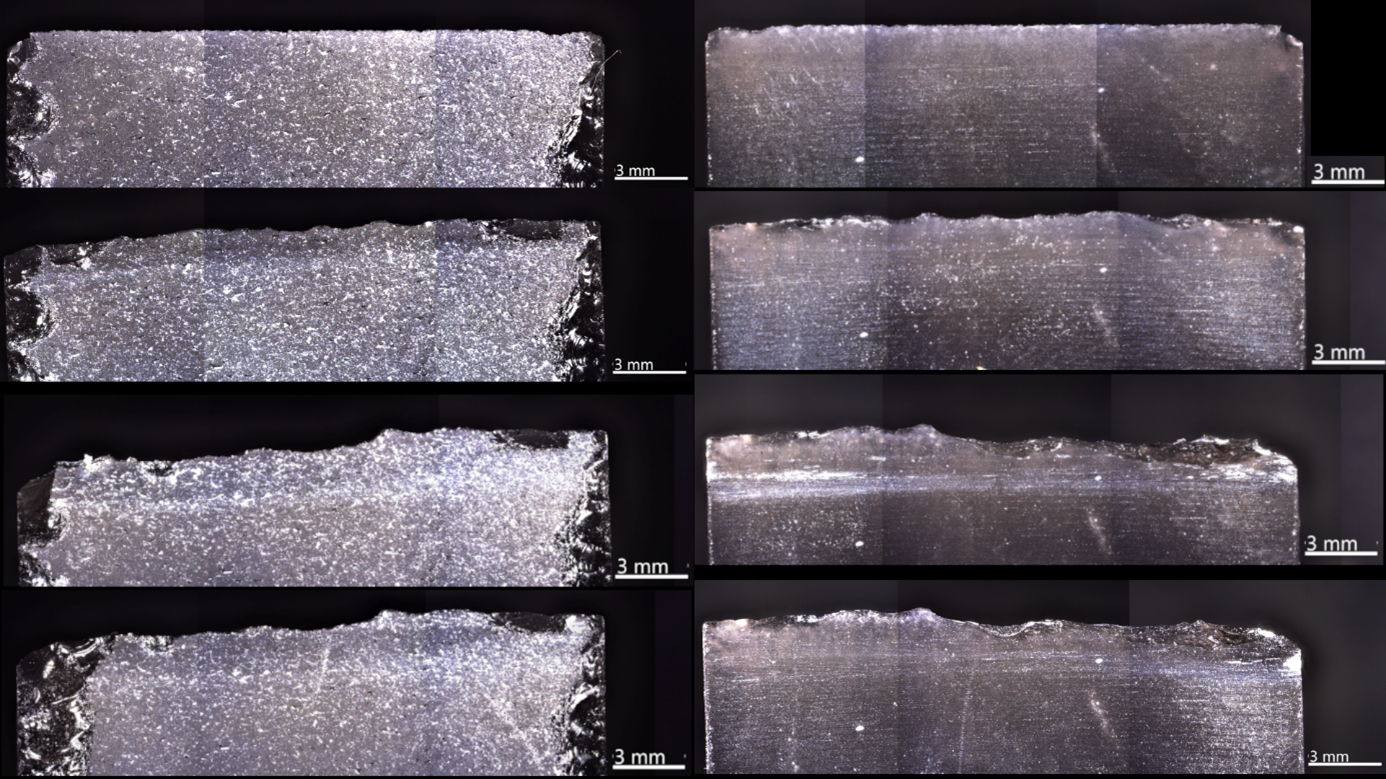

Supplement: SOM6 — (ZIP) [file pone.0327215.s006.zip › SOM_6_Zeiss_Smart_Zoom/ZEISS Smart zoom OBS4-5_ left_back_view_Right_front_view_top_ to_bottom_cycle_0_125_250_500.png]

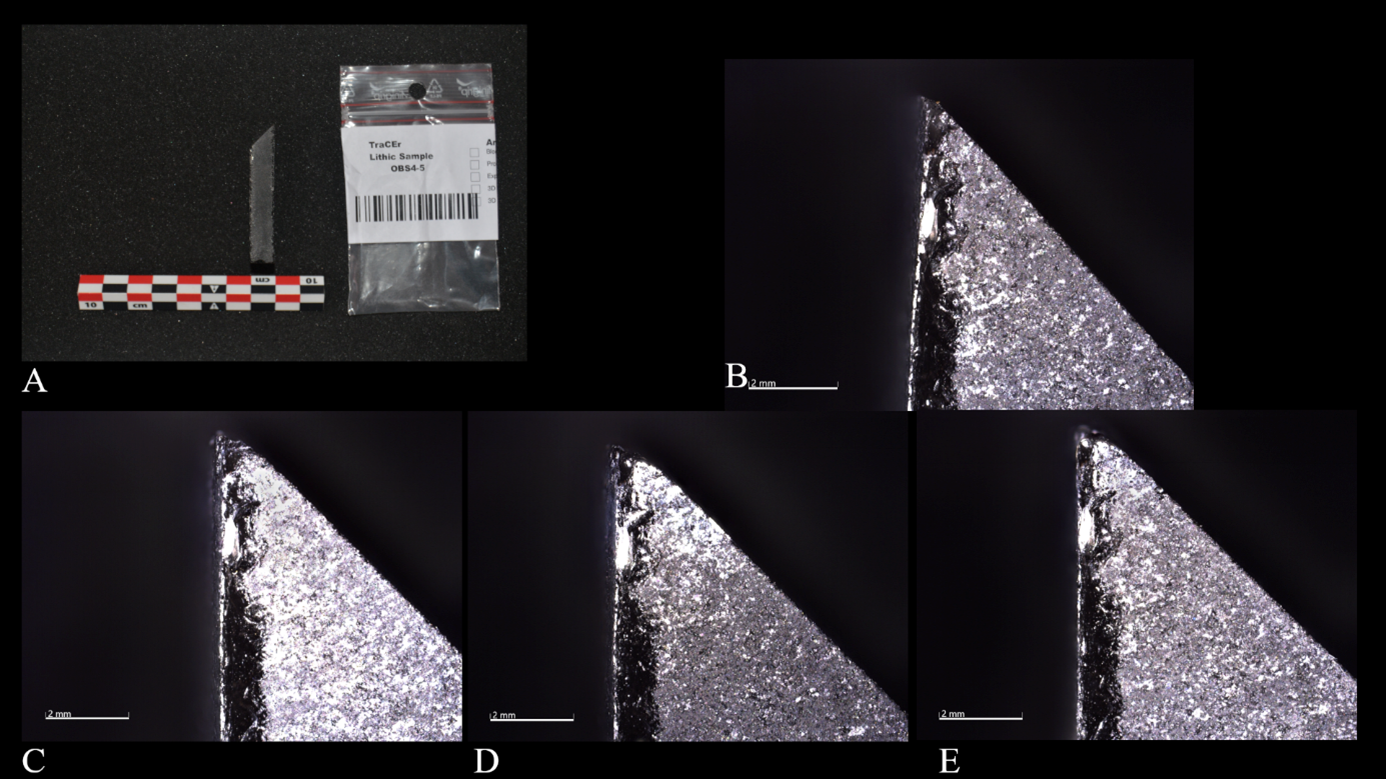

Supplement: SOM6 — (ZIP) [file pone.0327215.s006.zip › SOM_6_Zeiss_Smart_Zoom/ZEISS Smart zoom OBS4-5_ Profile view, A – Sample ID; B – cycle 0; C – cycle 125; D - cycle 250; E – cycle 500.png]

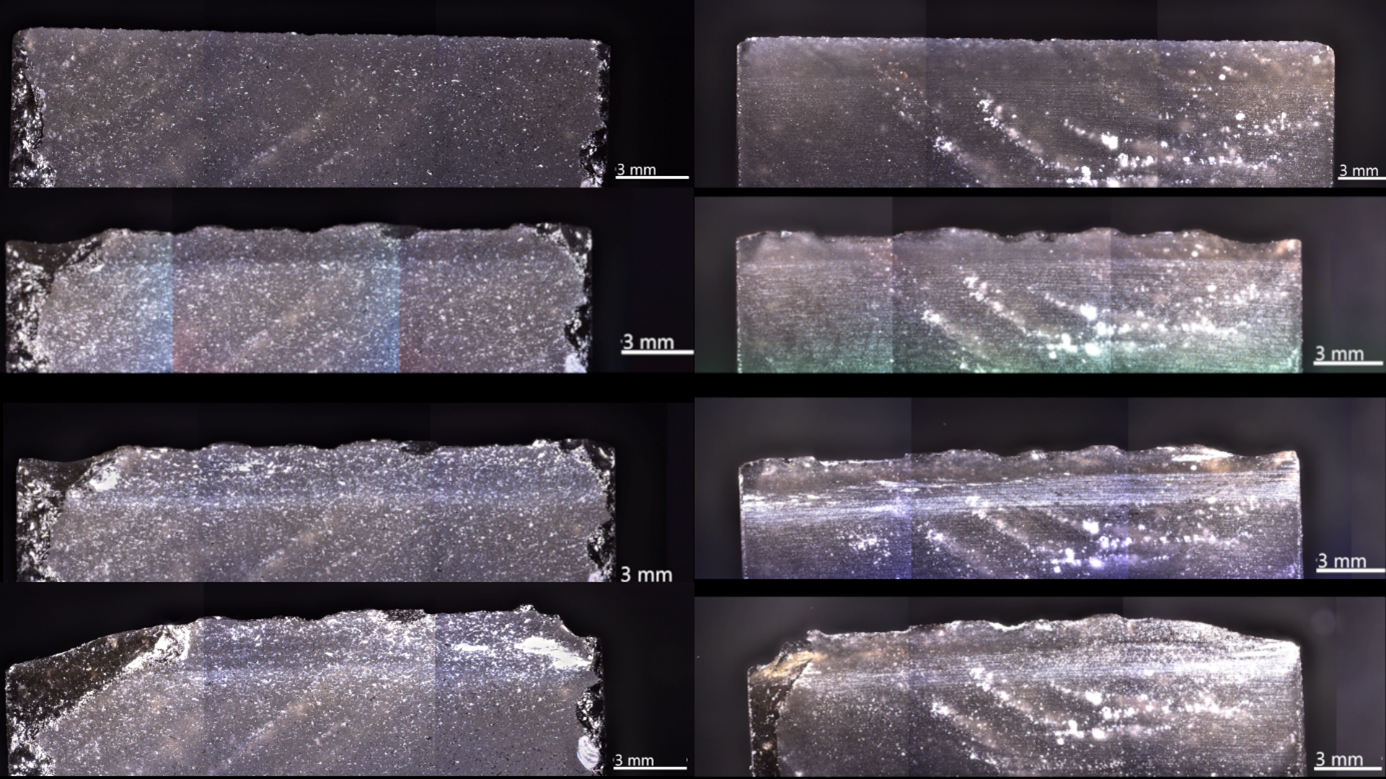

Supplement: SOM6 — (ZIP) [file pone.0327215.s006.zip › SOM_6_Zeiss_Smart_Zoom/ZEISS Smart zoom OBS4-6_left_back_view_Right_front_view_top_ to_bottom_cycle_0_125_250_500.png]

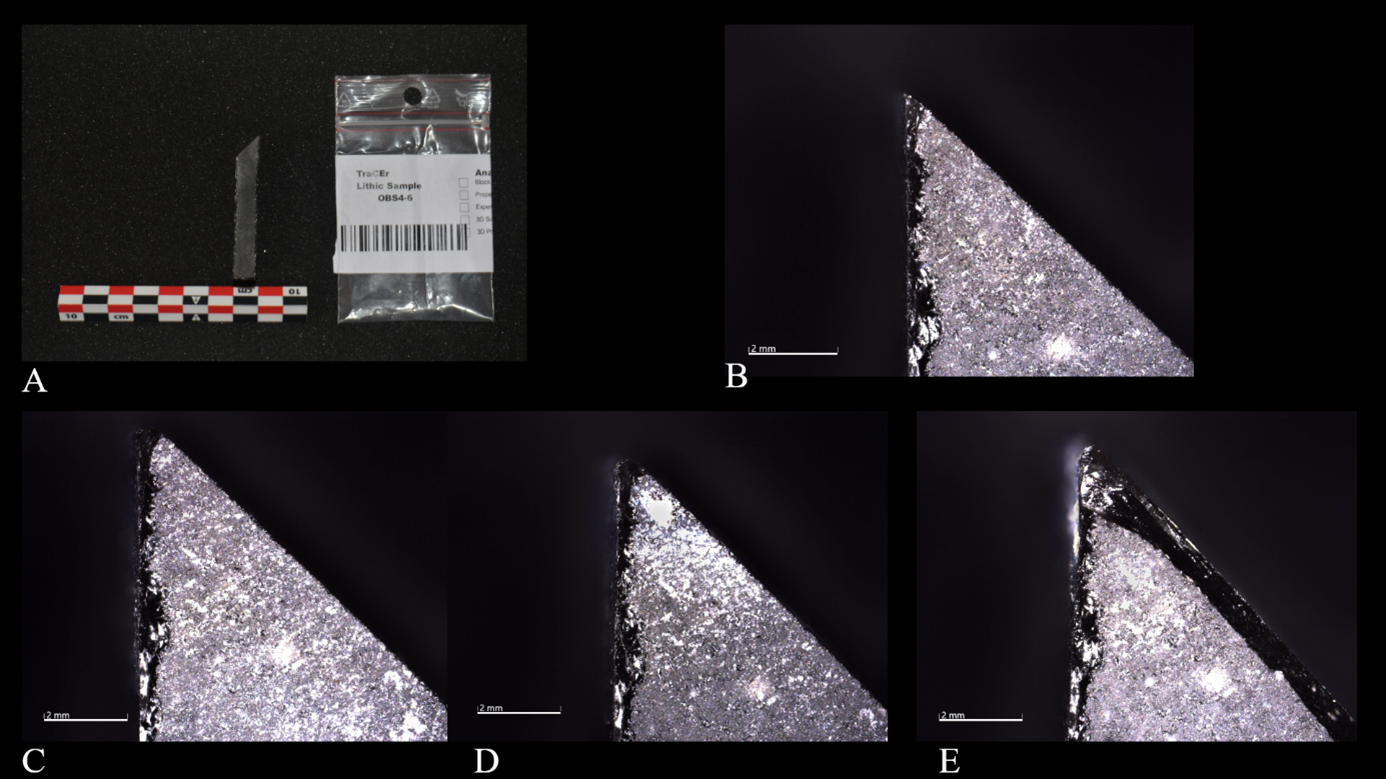

Supplement: SOM6 — (ZIP) [file pone.0327215.s006.zip › SOM_6_Zeiss_Smart_Zoom/ZEISS Smart zoom OBS4-6_Profile view, A – Sample ID; B – cycle 0; C – cycle 125; D -cycle 250; E –cycle 500.png]

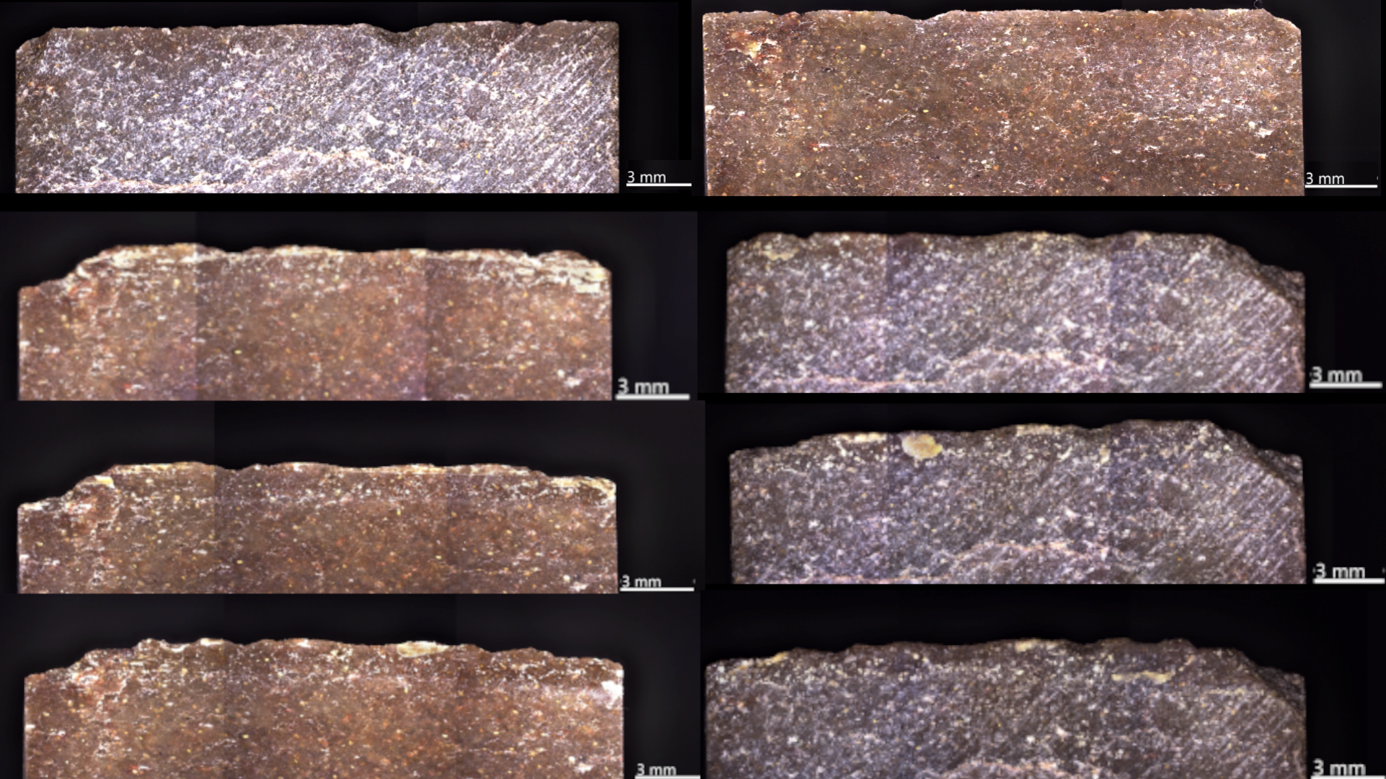

Supplement: SOM6 — (ZIP) [file pone.0327215.s006.zip › SOM_6_Zeiss_Smart_Zoom/ZEISS Smart zoom QTZ1-1_left_back_view_Right_front_view_top_ to_bottom_cycle_0_125_250_500.png]

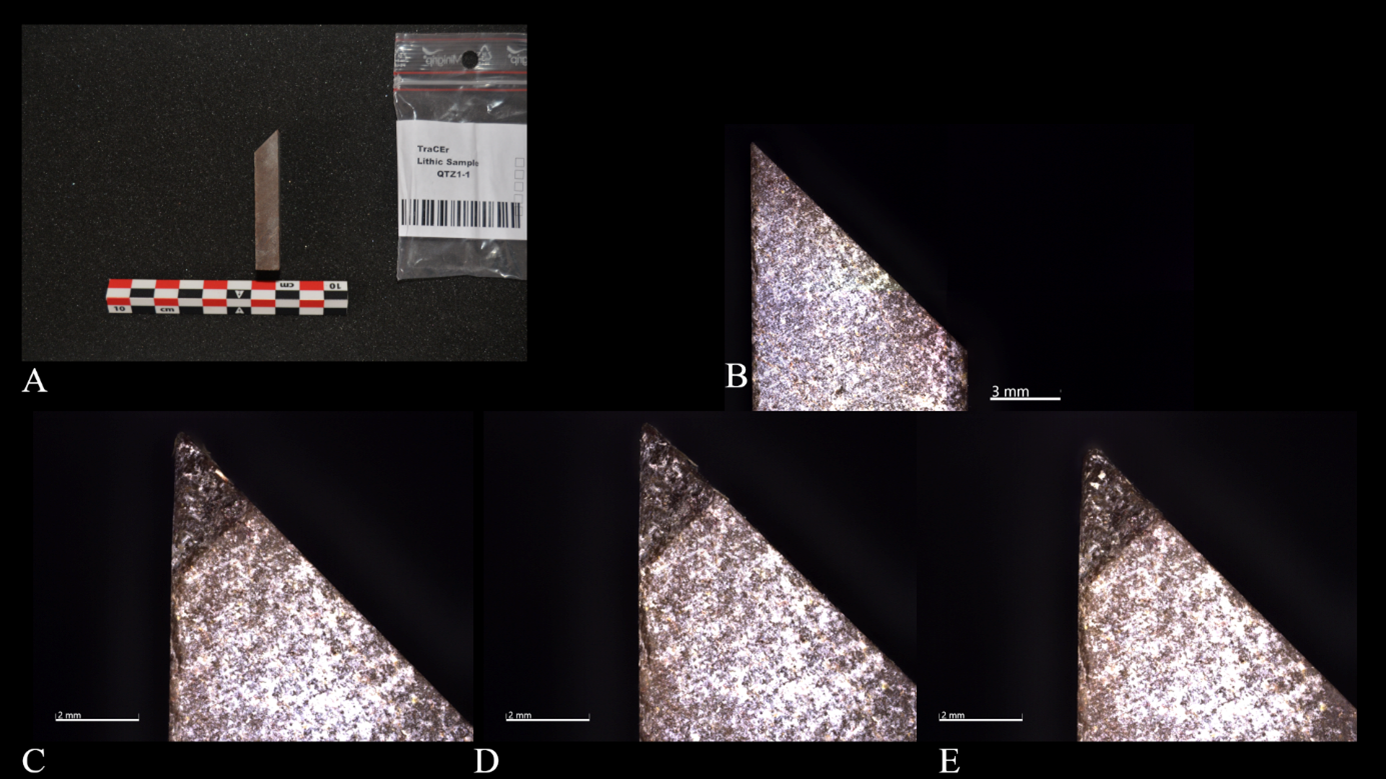

Supplement: SOM6 — (ZIP) [file pone.0327215.s006.zip › SOM_6_Zeiss_Smart_Zoom/ZEISS Smart zoom QTZ1-1_Profile view, A – Sample_ID; B – cycle 0; C –cycle 125; D -cycle 250; E –cycle 500.png]

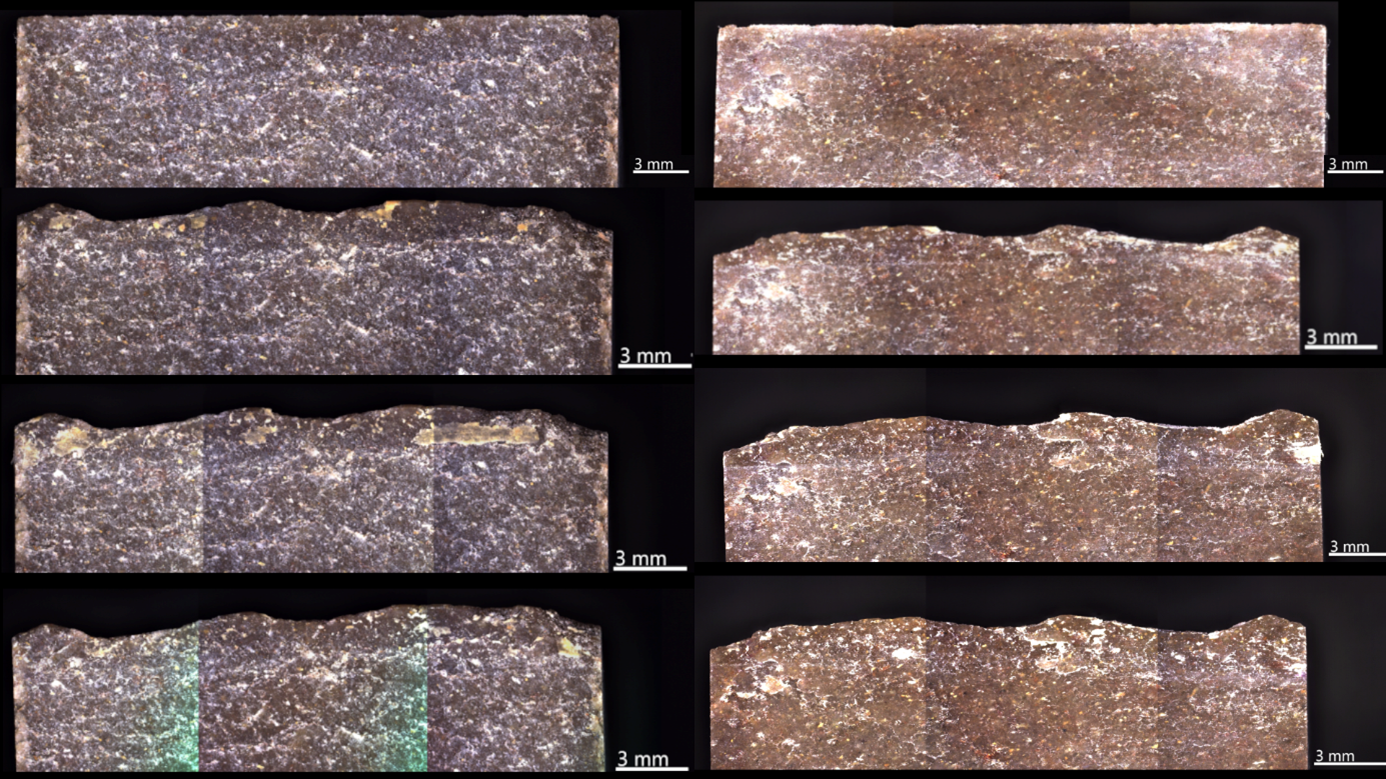

Supplement: SOM6 — (ZIP) [file pone.0327215.s006.zip › SOM_6_Zeiss_Smart_Zoom/ZEISS Smart zoom QTZ1-2_left_back_view_Right_front_view_top_ to_bottom_cycle_0_125_250_500.png]

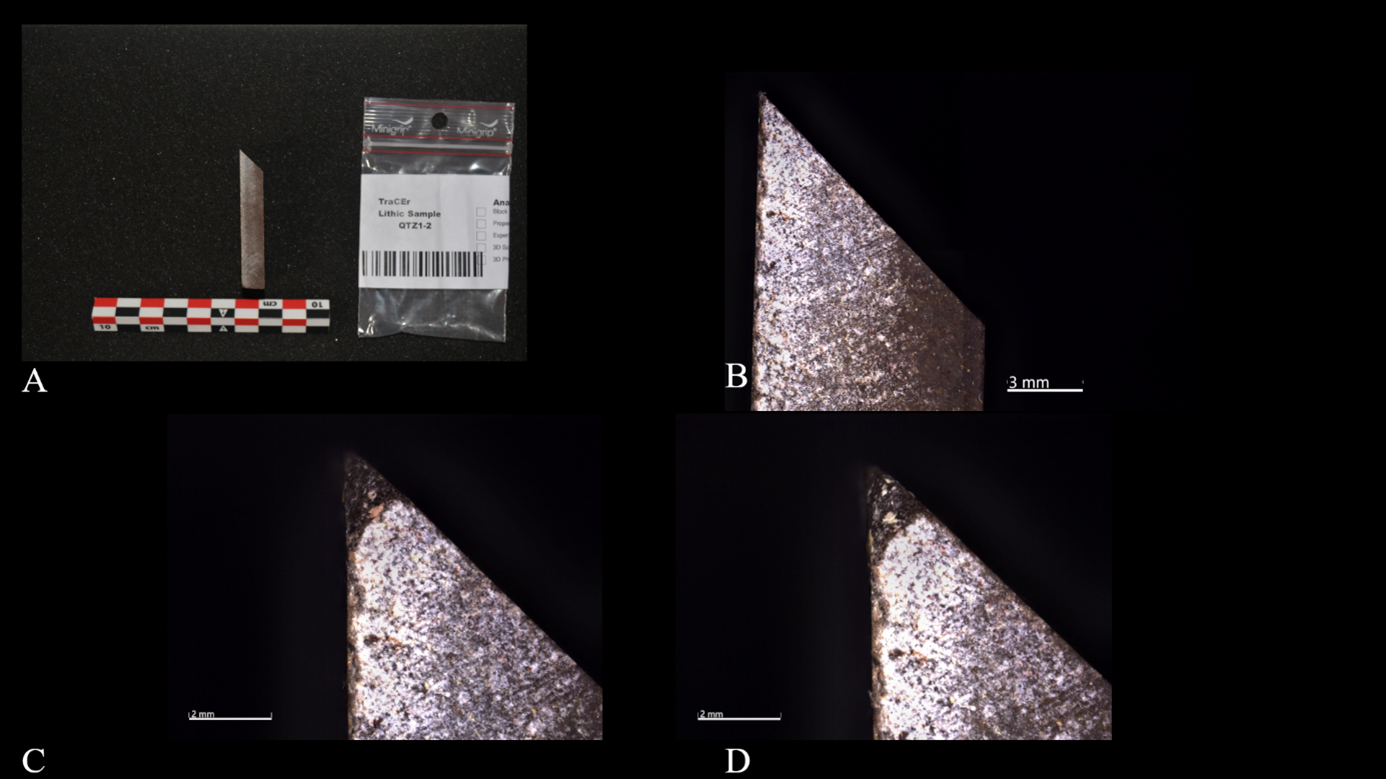

Supplement: SOM6 — (ZIP) [file pone.0327215.s006.zip › SOM_6_Zeiss_Smart_Zoom/ZEISS Smart zoom QTZ1-2_Profile view, A – Sample_ID_ B –cycle 0_ C –cycle 125_ D –cycle 500.png]

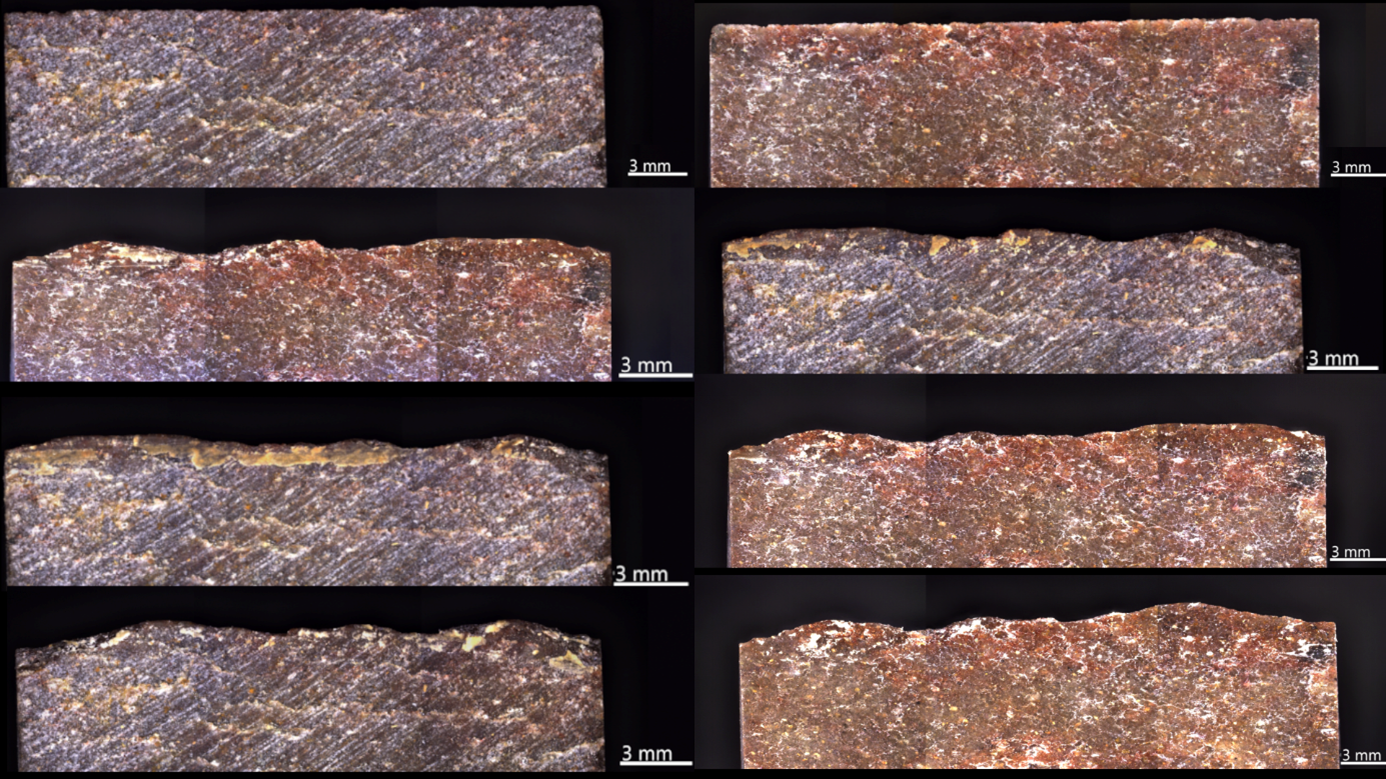

Supplement: SOM6 — (ZIP) [file pone.0327215.s006.zip › SOM_6_Zeiss_Smart_Zoom/ZEISS Smart zoom QTZ1-5_left_back_view_Right_front_view_top_ to_bottom_cycle_0_125_250_500.png]

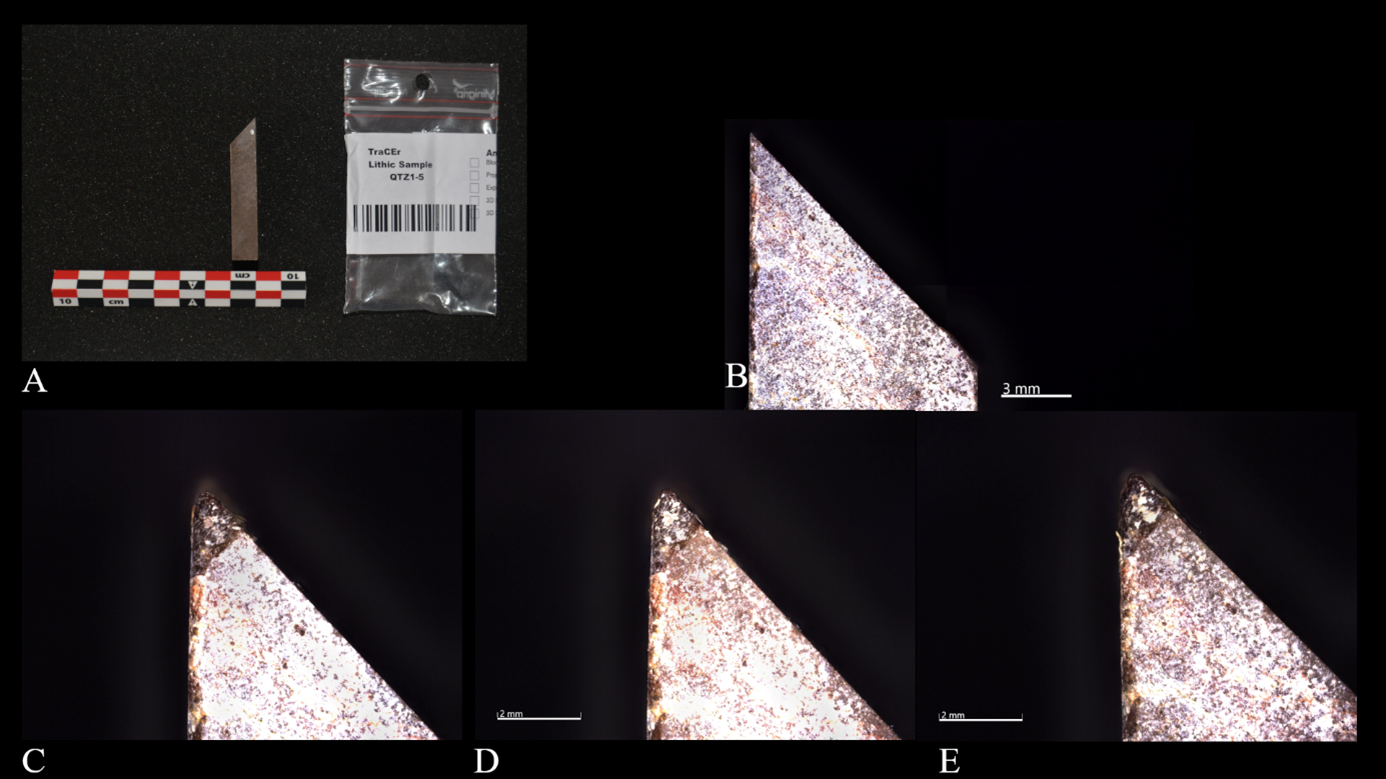

Supplement: SOM6 — (ZIP) [file pone.0327215.s006.zip › SOM_6_Zeiss_Smart_Zoom/ZEISS Smart zoom QTZ1-5_Profile view, A – Sample_ID; B –cycle 0; C –cycle 125; D - cycle 250; E –cycle 500.png]
