# Supplementary material for: The dichotomy of human decision-making: An experimental assessment of stone tool efficiency
Source: PLoS One. 2025 Jul 18;20(7):e0327215. doi: 10.1371/journal.pone.0327215 (PMC12273975; doi:10.1371/journal.pone.0327215)
Supplement: SOM7 — (ZIP) [file pone.0327215.s007.zip › SOM_7_R_scripts_data_process_and_analysis/analysis3d (1).html]

Plots 3D data


# Plots 3D data

#### David Nora, João Marreiros, Walter Gneisinger, Antonella Pedergnana, Telmo Pereira

#### 2024-10-29 13:59:48.29629

---

# Content

This script reads and plots the data from the 3D volume loss, which
was calculated using CloudCompare cloud-to-mesh distance tool. The knit
directory for this script is the project directory.

---

# Load packages

```
library(R.utils)
```

```
Warning: package 'R.utils' was built under R version 4.3.1
```

```
Warning: package 'R.oo' was built under R version 4.3.1
```

```
library(ggplot2)
```

```
Warning: package 'ggplot2' was built under R version 4.3.1
```

```
library(tools)
library(tidyverse)
```

```
Warning: package 'tidyr' was built under R version 4.3.1
```

```
Warning: package 'readr' was built under R version 4.3.1
```

```
Warning: package 'dplyr' was built under R version 4.3.1
```

```
Warning: package 'stringr' was built under R version 4.3.1
```

```
Warning: package 'lubridate' was built under R version 4.3.1
```

```
library(doBy)
```

```
Warning: package 'doBy' was built under R version 4.3.3
```

```
library(ggrepel)
```

```
Warning: package 'ggrepel' was built under R version 4.3.1
```

---

# Load data db

```
# Cloud-to-mesh calculations (before and after) for each sample were compiled in a single .csv file. File is formatted as exported from CloudCompare. We add a new column "Cycles".
# Import dataset
imp_data <- read_csv2("../rawdata/3dedge.csv")
str(imp_data)
```

```
spc_tbl_ [10,915 × 9] (S3: spec_tbl_df/tbl_df/tbl/data.frame)
 $ ID          : chr [1:10915] "DAC3-2" "DAC3-2" "DAC3-2" "DAC3-2" ...
 $ raw.material: chr [1:10915] "dacite" "dacite" "dacite" "dacite" ...
 $ grain       : chr [1:10915] "coarse" "coarse" "coarse" "coarse" ...
 $ cycle       : chr [1:10915] "0-125" "0-125" "0-125" "0-125" ...
 $ class       : num [1:10915] 1 2 3 4 5 6 7 8 9 10 ...
 $ value       : num [1:10915] 29 35 26 27 26 38 22 26 25 19 ...
 $ end         : chr [1:10915] "0.200000003" "0.201890059" "0.203780115" "0.205670171" ...
 $ start       : chr [1:10915] "0.201890059" "0.203780115" "0.205670171" "0.207560227" ...
 $ ...9        : logi [1:10915] NA NA NA NA NA NA ...
 - attr(*, "spec")=
  .. cols(
  ..   ID = col_character(),
  ..   raw.material = col_character(),
  ..   grain = col_character(),
  ..   cycle = col_character(),
  ..   class = col_double(),
  ..   value = col_double(),
  ..   end = col_character(),
  ..   start = col_character(),
  ..   ...9 = col_logical()
  .. )
 - attr(*, "problems")=<externalptr>
```

# Reorder raw material categories

```
imp_data$raw.material <- factor(imp_data$raw.material, levels=c('flint', 'obsidian', 'dacite', 'quartzite'))
```

# organise and sort data

```
# organise and represent data differently so that mean and sd can be calculated
# now it shows how many times a value (distance) is represented by each class (sample segmentation)

data.expanded <- imp_data[rep(row.names(imp_data), imp_data$value), 1:7]

## Organise and categorise cycles (numeric to categorical) and rock types
data.expanded <- data.expanded %>% mutate(Cyclecat = case_when(cycle >= 0  & cycle <= 124 ~ 'Stage 1 (0-125 cycles)',
                                             cycle >= 125  & cycle <= 250 ~ 'Stage 2 (126-250 cycles)',
                                             cycle >= 250  & cycle <= 500 ~ 'Stage 3 (251-500 cycles)'))

write_csv(data.expanded, "../deriveddata/dataexpanded.csv")
```

# Summarize data

```
# Calculate distance (edge reduction) means between raw materials, samples and cycles

## exclude 0-500 cycles, which corresponds to the starting and ending point
data.expanded.cycles <- data.expanded %>% filter(!cycle == "0-500")

## Calculate

volumelossstats <- data.expanded.cycles %>% group_by(ID, grain, raw.material, Cyclecat) %>%
      summarise(
      vlcount = n(),
      vlmax = max(value, na.rm = TRUE),
      vlmin = min (value, na.rm = TRUE),
      vlmean = mean(value, na.rm = TRUE),
      vlsd = sd(value, na.rm = TRUE),
      vlmedian = median(value, na.rm = TRUE),
   )

write_csv(volumelossstats, "../stats/volumelossstats.csv")
```

# Plot data organised by raw material and sample ID

```
volumelossstats$raw.material <- factor(volumelossstats$raw.material, levels=c('flint', 'obsidian', 'dacite', 'quartzite'))

volumelossstats$grain <- factor(volumelossstats$grain, levels=c("fine", "coarse"))

# Plot data (edge reduction) organised by raw material and cycle (sequential experiment: 0-125, 125-250, 250-500)

## Plot selected data (cycles)

edre <- ggplot(data = volumelossstats, aes(x = Cyclecat, y = vlmax)) + 
  geom_boxplot(aes(colour = raw.material), outlier.colour = "", outlier.shape = 1) +
  geom_jitter(aes(colour = raw.material), alpha=0.2, position=position_jitter(w=0.1,h=0.1)) +
  labs(y = "Edge wear (aHd)", x = "", colour = "Raw material")

ggsave("../plots/cycles_rawmaterial.png")

print(edre)
```

---

# sessionInfo() and RStudio version

```
sessionInfo()
```

```
R version 4.3.0 (2023-04-21)
Platform: aarch64-apple-darwin20 (64-bit)
Running under: macOS 15.0.1

Matrix products: default
BLAS:   /Library/Frameworks/R.framework/Versions/4.3-arm64/Resources/lib/libRblas.0.dylib 
LAPACK: /Library/Frameworks/R.framework/Versions/4.3-arm64/Resources/lib/libRlapack.dylib;  LAPACK version 3.11.0

locale:
[1] en_US.UTF-8/en_US.UTF-8/en_US.UTF-8/C/en_US.UTF-8/en_US.UTF-8

time zone: Europe/Lisbon
tzcode source: internal

attached base packages:
[1] tools     stats     graphics  grDevices utils     datasets  methods  
[8] base     

other attached packages:
 [1] ggrepel_0.9.5     doBy_4.6.22       lubridate_1.9.3   forcats_1.0.0    
 [5] stringr_1.5.1     dplyr_1.1.4       purrr_1.0.2       readr_2.1.5      
 [9] tidyr_1.3.1       tibble_3.2.1      tidyverse_2.0.0   ggplot2_3.5.1    
[13] R.utils_2.12.3    R.oo_1.26.0       R.methodsS3_1.8.2

loaded via a namespace (and not attached):
 [1] gtable_0.3.5          xfun_0.45             bslib_0.7.0          
 [4] lattice_0.22-6        tzdb_0.4.0            vctrs_0.6.5          
 [7] generics_0.1.3        parallel_4.3.0        fansi_1.0.6          
[10] highr_0.11            pkgconfig_2.0.3       Matrix_1.6-5         
[13] lifecycle_1.0.4       farver_2.1.2          compiler_4.3.0       
[16] textshaping_0.4.0     microbenchmark_1.4.10 munsell_0.5.1        
[19] htmltools_0.5.8.1     sass_0.4.9            yaml_2.3.9           
[22] pillar_1.9.0          crayon_1.5.3          jquerylib_0.1.4      
[25] MASS_7.3-60.0.1       cachem_1.1.0          boot_1.3-30          
[28] Deriv_4.1.3           tidyselect_1.2.1      digest_0.6.36        
[31] stringi_1.8.4         labeling_0.4.3        cowplot_1.1.3        
[34] fastmap_1.2.0         grid_4.3.0            colorspace_2.1-0     
[37] cli_3.6.3             magrittr_2.0.3        utf8_1.2.4           
[40] broom_1.0.6           withr_3.0.0           scales_1.3.0         
[43] backports_1.5.0       bit64_4.0.5           timechange_0.3.0     
[46] rmarkdown_2.27        modelr_0.1.11         bit_4.0.5            
[49] ragg_1.3.2            hms_1.1.3             evaluate_0.24.0      
[52] knitr_1.48            rlang_1.1.4           Rcpp_1.0.12          
[55] glue_1.7.0            rstudioapi_0.16.0     vroom_1.6.5          
[58] jsonlite_1.8.8        R6_2.5.1              systemfonts_1.1.0
```

---

END OF SCRIPT
