# Supplementary material for: The dichotomy of human decision-making: An experimental assessment of stone tool efficiency
Source: PLoS One. 2025 Jul 18;20(7):e0327215. doi: 10.1371/journal.pone.0327215 (PMC12273975; doi:10.1371/journal.pone.0327215)
Supplement: SOM7 — (ZIP) [file pone.0327215.s007.zip › SOM_7_R_scripts_data_process_and_analysis/analysishardness (1).html]

Plots Equotip Leeb rebound C hardness measurements


# Plots Equotip Leeb rebound C hardness measurements

#### David Nora, Joao Marreiros, Walter Gneisinger

#### 2024-03-20

---

# Content

This script reads and plots the data from the Leeb Rebound hardness.
Hardness was measured on each rock using the device Equotip 550 Leeb
Impact device C (HLC). For details on the methods and data acquisition,
please visit the Materials and Methods section of the paper.

The knit directory for this script is the project directory.

|  |
| --- |
| # Load packages |
| `r library(tidyverse)` |
| `## Warning: package 'ggplot2' was built under R version 4.3.1` |
| `## Warning: package 'readr' was built under R version 4.3.1` |
| `## Warning: package 'dplyr' was built under R version 4.3.1` |
| `## Warning: package 'stringr' was built under R version 4.3.1` |
| `## Warning: package 'lubridate' was built under R version 4.3.1` |
| `## ── Attaching core tidyverse packages ──────────────────────── tidyverse 2.0.0 ── ## ✔ dplyr 1.1.4 ✔ readr 2.1.5 ## ✔ forcats 1.0.0 ✔ stringr 1.5.1 ## ✔ ggplot2 3.4.4 ✔ tibble 3.2.1 ## ✔ lubridate 1.9.3 ✔ tidyr 1.3.0 ## ✔ purrr 1.0.2 ## ── Conflicts ────────────────────────────────────────── tidyverse_conflicts() ── ## ✖ dplyr::filter() masks stats::filter() ## ✖ dplyr::lag() masks stats::lag() ## ℹ Use the conflicted package (<http://conflicted.r-lib.org/>) to force all conflicts to become errors` |
| `r library(AICcmodavg)` |
| `## Warning: package 'AICcmodavg' was built under R version 4.3.1` |
| `r library(ggplot2) library(utils) library(qwraps2)` |
| `## Warning: package 'qwraps2' was built under R version 4.3.1` |
| `## ## Attaching package: 'qwraps2' ## ## The following object is masked from 'package:ggplot2': ## ## mean_se` |
| `r library(kableExtra)` |
| `## ## Attaching package: 'kableExtra' ## ## The following object is masked from 'package:dplyr': ## ## group_rows` |
| `r library(doBy)` |
| `## Warning: package 'doBy' was built under R version 4.3.1` |
| `## ## Attaching package: 'doBy' ## ## The following object is masked from 'package:dplyr': ## ## order_by` |
| `r library(ggpubr)` |
| `## ## Attaching package: 'ggpubr' ## ## The following objects are masked from 'package:qwraps2': ## ## mean_ci, mean_sd, median_iqr` |

# Read in original xlsx-file

```
# List all CSV files in dir_in

harddata <- read_csv2("../rawdata/data.csv")
```

```
## ℹ Using "','" as decimal and "'.'" as grouping mark. Use `read_delim()` for more control.
```

```
## Rows: 12 Columns: 15
## ── Column specification ────────────────────────────────────────────────────────
## Delimiter: ";"
## chr   (4): ID, rawmaterial, grain, Date
## dbl  (10): M1, M2, M3, M4, M5, M6, M7, M8, M9, M10
## time  (1): Time
## 
## ℹ Use `spec()` to retrieve the full column specification for this data.
## ℹ Specify the column types or set `show_col_types = FALSE` to quiet this message.
```

```
# A total of 10 measurements were taken on each rock sample, 4 raw materials, 6 samples each
```

# Organise data

```
# organising and summarising data by Sample ID
longdata <- harddata %>%
  gather("M1", "M2","M3", "M4", "M5","M6","M7", "M8", "M9", "M10", key = Measurment, value = HLC)

# Show new format
longdata
```

```
## # A tibble: 120 × 7
##    ID      rawmaterial grain  Date       Time     Measurment   HLC
##    <chr>   <chr>       <chr>  <chr>      <time>   <chr>      <dbl>
##  1 QTZ1-5  quartzite   coarse 09/06/2021 14:22:10 M1           919
##  2 QTZ1-2  quartzite   coarse 09/06/2021 14:12:22 M1           863
##  3 QTZ1-1  quartzite   coarse 09/06/2021 14:05:18 M1           910
##  4 OBS4-6  obsidian    fine   09/06/2021 13:59:22 M1           927
##  5 OBS4-5  obsidian    fine   09/06/2021 13:51:50 M1           969
##  6 OBS4-4  obsidian    fine   09/06/2021 13:48:41 M1           966
##  7 FLT10-6 flint       fine   09/06/2021 12:22:13 M1           954
##  8 FLT10-5 flint       fine   09/06/2021 12:18:19 M1           963
##  9 FLT10-2 flint       fine   09/06/2021 12:00:33 M1           968
## 10 DAC3-6  dacite      coarse 09/06/2021 15:03:51 M1           966
## # ℹ 110 more rows
```

```
# Save the new format
write_csv(longdata, "../deriveddata/longdata.csv")
```

# Descriptive stats

```
# descriptive statistics
# summarise data by each sample

# summarise by "Raw Material" but keep other columns

hlcstatsmaterial <- longdata %>% group_by(rawmaterial) %>%
      summarise(
      hlccount = n(),
      hlcmax = max(HLC, na.rm = TRUE),
      hlcmin = min (HLC, na.rm = TRUE),
      hlcmean = mean(HLC, na.rm = TRUE),
      hlcsd = sd(HLC, na.rm = TRUE),
      hlcmedian = median(HLC, na.rm = TRUE),
  ) 

# summarise by "Sample/ID" but keep other columns

hlcstatssample <- longdata %>% group_by(ID, rawmaterial) %>%
      summarise(
      vlcount = n(),
      vlmax = max(HLC, na.rm = TRUE),
      vlmin = min (HLC, na.rm = TRUE),
      vlmean = mean(HLC, na.rm = TRUE),
      vlsd = sd(HLC, na.rm = TRUE),
      vlmedian = median(HLC, na.rm = TRUE),
   )
```

```
## `summarise()` has grouped output by 'ID'. You can override using the `.groups`
## argument.
```

```
# View results
hlcstatsmaterial
```

```
## # A tibble: 4 × 7
##   rawmaterial hlccount hlcmax hlcmin hlcmean hlcsd hlcmedian
##   <chr>          <int>  <dbl>  <dbl>   <dbl> <dbl>     <dbl>
## 1 dacite            30    975    865    954. 23.9       964 
## 2 flint             30    972    900    949  20.6       954.
## 3 obsidian          30    970    927    959.  9.27      960.
## 4 quartzite         30    935    850    897. 23.3       897
```

```
hlcstatssample
```

```
## # A tibble: 12 × 8
## # Groups:   ID [12]
##    ID      rawmaterial vlcount vlmax vlmin vlmean  vlsd vlmedian
##    <chr>   <chr>         <int> <dbl> <dbl>  <dbl> <dbl>    <dbl>
##  1 DAC3-2  dacite           10   962   865   932. 28.7      942 
##  2 DAC3-4  dacite           10   972   955   965.  6.04     968.
##  3 DAC3-6  dacite           10   975   931   964. 14.4      968.
##  4 FLT10-2 flint            10   972   953   966.  5.72     968 
##  5 FLT10-5 flint            10   968   900   936  22.0      934.
##  6 FLT10-6 flint            10   961   904   945. 18.4      952 
##  7 OBS4-4  obsidian         10   970   959   966.  3.59     966 
##  8 OBS4-5  obsidian         10   969   953   960.  5.90     960.
##  9 OBS4-6  obsidian         10   962   927   952. 11.0      956.
## 10 QTZ1-1  quartzite        10   910   855   883. 19.1      890 
## 11 QTZ1-2  quartzite        10   935   863   908. 21.5      914.
## 12 QTZ1-5  quartzite        10   929   850   899. 24.0      903
```

```
# Same results
write_csv(hlcstatsmaterial, "../stats/stats_hardness_maerial.csv")
write_csv(hlcstatssample, "../stats/stats_hardness_sample.csv")
```

# Reorder raw material categories

```
longdata$rawmaterial <- factor(longdata$rawmaterial, levels=c('flint', 'obsidian', 'dacite', 'quartzite'))
```

# Plot data (one numerical continuious variable - HLC)

```
# Boxplot

bp <- ggplot (longdata, aes(rawmaterial, HLC, color = grain)) + 
  geom_jitter(aes(shape = rawmaterial)) + 
  labs(x="Raw material", y="Leeb Rebound Hardness in HLC", title="", color="Category", shape="Raw material") +
  geom_boxplot()
             

print(bp)
```

```
ggsave("../plots/hardnessplot.png")
```

```
## Saving 7 x 5 in image
```

```
# Density plot
dp <- ggplot (longdata, aes(x = HLC, color = grain, linetype = rawmaterial)) + 
             theme_classic() +
  scale_linetype_manual(values=c("twodash", "dotted", "solid", "longdash")) +
             geom_density() + labs(x="Leeb Rebound Hardness in HLC", y="Density", title="")

print(dp)
```

```
ggsave("../plots/densityplot.png")
```

```
## Saving 7 x 5 in image
```

# sessionInfo() and RStudio version

```
sessionInfo()
```

```
## R version 4.3.0 (2023-04-21)
## Platform: aarch64-apple-darwin20 (64-bit)
## Running under: macOS 14.4
## 
## Matrix products: default
## BLAS:   /Library/Frameworks/R.framework/Versions/4.3-arm64/Resources/lib/libRblas.0.dylib 
## LAPACK: /Library/Frameworks/R.framework/Versions/4.3-arm64/Resources/lib/libRlapack.dylib;  LAPACK version 3.11.0
## 
## locale:
## [1] en_US.UTF-8/en_US.UTF-8/en_US.UTF-8/C/en_US.UTF-8/en_US.UTF-8
## 
## time zone: Europe/Lisbon
## tzcode source: internal
## 
## attached base packages:
## [1] stats     graphics  grDevices utils     datasets  methods   base     
## 
## other attached packages:
##  [1] ggpubr_0.6.0.999 doBy_4.6.20      kableExtra_1.3.4 qwraps2_0.6.0   
##  [5] AICcmodavg_2.3-3 lubridate_1.9.3  forcats_1.0.0    stringr_1.5.1   
##  [9] dplyr_1.1.4      purrr_1.0.2      readr_2.1.5      tidyr_1.3.0     
## [13] tibble_3.2.1     ggplot2_3.4.4    tidyverse_2.0.0 
## 
## loaded via a namespace (and not attached):
##  [1] gtable_0.3.4          xfun_0.41             bslib_0.6.1          
##  [4] rstatix_0.7.2         lattice_0.22-5        tzdb_0.4.0           
##  [7] vctrs_0.6.5           tools_4.3.0           generics_0.1.3       
## [10] stats4_4.3.0          parallel_4.3.0        fansi_1.0.6          
## [13] highr_0.10            pkgconfig_2.0.3       Matrix_1.6-5         
## [16] webshot_0.5.5         lifecycle_1.0.4       farver_2.1.1         
## [19] compiler_4.3.0        textshaping_0.3.7     microbenchmark_1.4.10
## [22] munsell_0.5.0         carData_3.0-5         htmltools_0.5.7      
## [25] sass_0.4.8            yaml_2.3.8            crayon_1.5.2         
## [28] car_3.1-2             pillar_1.9.0          jquerylib_0.1.4      
## [31] MASS_7.3-60.0.1       cachem_1.0.8          abind_1.4-5          
## [34] nlme_3.1-164          Deriv_4.1.3           tidyselect_1.2.0     
## [37] rvest_1.0.3           digest_0.6.34         stringi_1.8.3        
## [40] labeling_0.4.3        splines_4.3.0         fastmap_1.1.1        
## [43] grid_4.3.0            colorspace_2.1-0      cli_3.6.2            
## [46] magrittr_2.0.3        survival_3.5-7        utf8_1.2.4           
## [49] broom_1.0.5           withr_3.0.0           backports_1.4.1      
## [52] scales_1.3.0          bit64_4.0.5           unmarked_1.4.1       
## [55] timechange_0.2.0      rmarkdown_2.25        httr_1.4.7           
## [58] bit_4.0.5             ggsignif_0.6.4        ragg_1.2.7           
## [61] hms_1.1.3             VGAM_1.1-9            evaluate_0.23        
## [64] knitr_1.45            viridisLite_0.4.2     rlang_1.1.3          
## [67] Rcpp_1.0.12           xtable_1.8-4          glue_1.7.0           
## [70] xml2_1.3.6            vroom_1.6.5           svglite_2.1.3        
## [73] rstudioapi_0.15.0     jsonlite_1.8.8        R6_2.5.1             
## [76] systemfonts_1.0.5
```
