# Supplementary material for: The dichotomy of human decision-making: An experimental assessment of stone tool efficiency
Source: PLoS One. 2025 Jul 18;20(7):e0327215. doi: 10.1371/journal.pone.0327215 (PMC12273975; doi:10.1371/journal.pone.0327215)
Supplement: SOM7 — (ZIP) [file pone.0327215.s007.zip › SOM_7_R_scripts_data_process_and_analysis/relationship.html]

Relationship betwen variables


# Relationship betwen variables

#### David Nora, João Marreiros, Walter Gneisinger, Antonella Pedergnana, Telmo Pereira

#### 2024-10-29 14:13:05.071269

---

# Goal of the script

This script analysis the relationship between the different
variables. The knit directory for this script is the project
directory.

---

# Load packages

```
library(R.utils)
```

```
Warning: package 'R.utils' was built under R version 4.3.1
```

```
Warning: package 'R.oo' was built under R version 4.3.1
```

```
library(ggplot2)
```

```
Warning: package 'ggplot2' was built under R version 4.3.1
```

```
library(tools)
library(tidyverse)
```

```
Warning: package 'tidyr' was built under R version 4.3.1
```

```
Warning: package 'readr' was built under R version 4.3.1
```

```
Warning: package 'dplyr' was built under R version 4.3.1
```

```
Warning: package 'stringr' was built under R version 4.3.1
```

```
Warning: package 'lubridate' was built under R version 4.3.1
```

```
library(ggpubr)
```

---

# Load data db

```
# Derived data for each sample on penetration depth, leeb rebound hardness and edge reduction, were combined in a single .csv file

# Import dataset
imp_data <- read_csv2("../rawdata/data.csv")
str(imp_data)
```

```
spc_tbl_ [36 × 10] (S3: spec_tbl_df/tbl_df/tbl/data.frame)
 $ rawmaterial: chr [1:36] "Dacite" "Dacite" "Dacite" "Dacite" ...
 $ grain      : chr [1:36] "coarse" "coarse" "coarse" "coarse" ...
 $ cycle      : chr [1:36] "0-125" "125-250" "250-500" "0-125" ...
 $ sampleid   : chr [1:36] "DAC3-2" "DAC3-2" "DAC3-2" "DAC3-4" ...
 $ force      : num [1:36] -58.7 -58.4 -58.6 -58.5 -58 ...
 $ friction   : num [1:36] -13.166 -14.322 -0.894 9.873 -4.877 ...
 $ velocity   : num [1:36] 1.993 0.285 -0.126 2.658 1.406 ...
 $ depth      : num [1:36] 0.921 1.202 0.729 1.455 2.501 ...
 $ ahd        : num [1:36] 38 23 14 8 72 13 48 1 21 41 ...
 $ hlc        : num [1:36] 942 942 942 965 965 ...
 - attr(*, "spec")=
  .. cols(
  ..   rawmaterial = col_character(),
  ..   grain = col_character(),
  ..   cycle = col_character(),
  ..   sampleid = col_character(),
  ..   force = col_double(),
  ..   friction = col_double(),
  ..   velocity = col_double(),
  ..   depth = col_double(),
  ..   ahd = col_double(),
  ..   hlc = col_double()
  .. )
 - attr(*, "problems")=<externalptr>
```

# Reorder raw material categories

```
imp_data$rawmaterial <- factor(imp_data$rawmaterial, levels=c('Flint', 'Obsidian', 'Dacite', 'Quartzite'))
```

## Organise and categorise cycles (numeric to categorical) and rock types

```
imp_data <- imp_data %>% mutate(Cyclecat = case_when(cycle >= 0  & cycle <= 124 ~ 'Stage 1 (0-125 cycles)',
                                             cycle >= 125  & cycle <= 250 ~ 'Stage 2 (126-250 cycles)',
                                             cycle >= 250  & cycle <= 500 ~ 'Stage 3 (251-500 cycles)'))
```

# Plot and explore data

```
# Inspect dataset, check relationships

#depth vs hardness (scatter plot)
scat1 <- ggplot(imp_data,aes(hlc, depth, color = rawmaterial))+
  geom_point() +
  stat_conf_ellipse() +
  labs(y = "Penetration depth (mm)", x = "Hardness (HLC)", colour = "Raw Material")


print(scat1)
```

```
ggsave("../plots/hlcdepth.png")

#edge attrition vs hardness (scatter plot)
scat2 <- ggplot(imp_data,aes(hlc, ahd, color = rawmaterial)) + 
  geom_point() +
  stat_conf_ellipse() +
  labs(y = "Edge wear (aHd)", x = "Hardness (HLC)", colour = "Raw material")


print(scat2)
```

```
ggsave("../plots/hlcahd.png")

#edge attrition vs depth (scatter plot), not sure this one makes sense
scat3 <- ggplot(imp_data,aes(ahd, depth, color = rawmaterial)) + 
  geom_point() +
  stat_conf_ellipse() + 
  labs(y = "Penetration depth (mm)", x = "Edge wear (aHd)", colour = "Raw material")


print(scat3)
```

```
ggsave("../plots/ahddepth.png")

# arrange plots in a single figure
arranged <- ggarrange(scat1, scat2, common.legend = TRUE, ncol = 2, nrow = 1, font.label = list(size=10))

print(arranged)
```

```
ggsave("../plots/arranged.jpg", width = 8, height = 4)

#Efficiency (scatter plot effectiveness vs edge attrition, organized by cycle)
# efficiency = (depth / edge attrition)

scat4 <- ggplot(imp_data,aes(ahd, depth, color = rawmaterial)) + 
  geom_point() +
  facet_grid(. ~ Cyclecat) + 
  stat_conf_ellipse() + 
  labs(y = "Penetration depth (mm)", x = "Edge wear (aHd)", colour = "Raw material")

print(scat4)
```

```
ggsave("../plots/cyle.png")

scat5 <- ggplot(imp_data,aes(ahd, depth, color = rawmaterial)) + 
  geom_point() +
  stat_conf_ellipse() +
  labs(y = "Penetration depth (mm)", x = "Edge wear (aHd)", colour = "Raw material")

print(scat5)
```

```
ggsave("../plots/total.png")
```

---

# sessionInfo() and RStudio version

```
sessionInfo()
```

```
R version 4.3.0 (2023-04-21)
Platform: aarch64-apple-darwin20 (64-bit)
Running under: macOS 15.0.1

Matrix products: default
BLAS:   /Library/Frameworks/R.framework/Versions/4.3-arm64/Resources/lib/libRblas.0.dylib 
LAPACK: /Library/Frameworks/R.framework/Versions/4.3-arm64/Resources/lib/libRlapack.dylib;  LAPACK version 3.11.0

locale:
[1] en_US.UTF-8/en_US.UTF-8/en_US.UTF-8/C/en_US.UTF-8/en_US.UTF-8

time zone: Europe/Lisbon
tzcode source: internal

attached base packages:
[1] tools     stats     graphics  grDevices utils     datasets  methods  
[8] base     

other attached packages:
 [1] ggpubr_0.6.0.999  lubridate_1.9.3   forcats_1.0.0     stringr_1.5.1    
 [5] dplyr_1.1.4       purrr_1.0.2       readr_2.1.5       tidyr_1.3.1      
 [9] tibble_3.2.1      tidyverse_2.0.0   ggplot2_3.5.1     R.utils_2.12.3   
[13] R.oo_1.26.0       R.methodsS3_1.8.2

loaded via a namespace (and not attached):
 [1] gtable_0.3.5      xfun_0.45         bslib_0.7.0       rstatix_0.7.2    
 [5] tzdb_0.4.0        vctrs_0.6.5       generics_0.1.3    parallel_4.3.0   
 [9] fansi_1.0.6       highr_0.11        pkgconfig_2.0.3   lifecycle_1.0.4  
[13] compiler_4.3.0    farver_2.1.2      textshaping_0.4.0 munsell_0.5.1    
[17] carData_3.0-5     htmltools_0.5.8.1 sass_0.4.9        yaml_2.3.9       
[21] pillar_1.9.0      car_3.1-2         crayon_1.5.3      jquerylib_0.1.4  
[25] cachem_1.1.0      abind_1.4-5       tidyselect_1.2.1  digest_0.6.36    
[29] stringi_1.8.4     labeling_0.4.3    cowplot_1.1.3     fastmap_1.2.0    
[33] grid_4.3.0        colorspace_2.1-0  cli_3.6.3         magrittr_2.0.3   
[37] utf8_1.2.4        broom_1.0.6       withr_3.0.0       scales_1.3.0     
[41] backports_1.5.0   bit64_4.0.5       timechange_0.3.0  rmarkdown_2.27   
[45] bit_4.0.5         gridExtra_2.3     ggsignif_0.6.4    ragg_1.3.2       
[49] hms_1.1.3         evaluate_0.24.0   knitr_1.48        rlang_1.1.4      
[53] glue_1.7.0        rstudioapi_0.16.0 vroom_1.6.5       jsonlite_1.8.8   
[57] R6_2.5.1          systemfonts_1.1.0
```

---

END OF SCRIPT
